# Control of cotton fibre elongation by a homeodomain transcription factor GhHOX3

**DOI:** 10.1038/ncomms6519

**Published:** 2014-11-21

**Authors:** Chun-Min Shan, Xiao-Xia Shangguan, Bo Zhao, Xiu-Fang Zhang, Lu-men Chao, Chang-Qing Yang, Ling-Jian Wang, Hua-Yu Zhu, Yan-Da Zeng, Wang-Zhen Guo, Bao-Liang Zhou, Guan-Jing Hu, Xue-Ying Guan, Z. Jeffrey Chen, Jonathan F. Wendel, Tian-Zhen Zhang, Xiao-Ya Chen

**Affiliations:** 1 National Key Laboratory of Plant Molecular Genetics, National Plant Gene Research Center, Institute of Plant Physiology and Ecology, Shanghai Institutes for Biological Sciences, Chinese Academy of Sciences, Shanghai 200032, China; 2 National Key Laboratory of Crop Genetics & Germplasm Enhancement, MOE Hybrid Cotton R&D Engineering Research Center, Nanjing Agricultural University, Nanjing 210095, China; 3 Department of Ecology, Evolution and Organismal Biology, Iowa State University, Ames, Iowa 50011, USA; 4 Department of Molecular Biosciences, Center for Computational Biology and Bioinformatics, and Institute for Cellular and Molecular Biology, The University of Texas at Austin, Austin, Texas 78712, USA; 5 Plant Science Research Center, Shanghai Chenshan Botanical Garden, Shanghai 201602, China

## Abstract

Cotton fibres are unusually long, single-celled epidermal seed trichomes and a model for plant cell growth, but little is known about the regulation of fibre cell elongation. Here we report that a homeodomain-leucine zipper (HD-ZIP) transcription factor, GhHOX3, controls cotton fibre elongation. *GhHOX3* genes are localized to the 12th homoeologous chromosome set of allotetraploid cotton cultivars, associated with quantitative trait loci (QTLs) for fibre length. Silencing of *GhHOX3* greatly reduces (>80%) fibre length, whereas its overexpression leads to longer fibre. Combined transcriptomic and biochemical analyses identify target genes of GhHOX3 that also contain the L1-box *cis*-element, including two cell wall loosening protein genes *GhRDL1* and *GhEXPA1*. GhHOX3 interacts with GhHD1, another homeodomain protein, resulting in enhanced transcriptional activity, and with cotton DELLA, GhSLR1, repressor of the growth hormone gibberellin (GA). GhSLR1 interferes with the GhHOX3–GhHD1 interaction and represses target gene transcription. Our results uncover a novel mechanism whereby a homeodomain protein transduces GA signal to promote fibre cell elongation.

## Abstract (web summary)

Cotton fibre is the most important renewable material for textiles, with a huge economic output. Here the authors show that a homeodomain-leucine zipper transcription factor, GhHOX3, transduces a gibberellin signal that in turn promotes fibre cell elongation.

## Graphical abstract

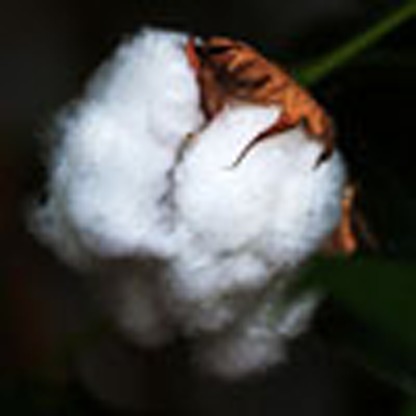

Cotton is widely grown in over 80 countries with an annual output value of $12 billion, mainly for cotton fibre, the most important natural and renewable material for the textile industry^1^. Fibre length is a key trait determining cotton quality and yield. In addition, the extensively elongated fibre cells, which undergo semi-synchronized development and rapid growth, provide an excellent system for the study of cell elongation^2^. Fibre development can be divided into four overlapping stages: initiation, elongation, secondary cell wall biosynthesis and maturation^3^. Although progress has been made in the identification of regulators controlling cotton fibre initiation (GhMYB25 and GhMYB25-like)^4^,^5^, and a number of factors have been proposed to affect fibre cell growth^6^,^7^,^8^,^9^,^10^,^11^, to date the key regulators of fibre elongation have not been identified, nor has the regulatory mechanism been elucidated.

Here we show that a homeodomain-leucine zipper (HD-ZIP) transcription factor, GhHOX3, plays a central role in controlling cotton fibre elongation. We also elucidate a molecular link between the phytohormone gibberellin (GA) and the homeobox regulators in promoting plant cell growth.

## Results

### *GhHOX3* associates with cotton fibre traits

The two widely cultivated cotton species, *Gossypium hirsutum* (AD)_1_ and *G. barbadense* (AD)_2_, are allotetraploids containing A- and D-homoeologous genomes, and among the diploids only the A-genome species produce spinnable fibre^12^. In *Arabidopsis thaliana*, the HD-ZIP IV subfamily factor GLABRA2 (AtGL2) is a positive regulator of the trichome development, functioning downstream of the initiation complex^13^. We previously reported that three HD-ZIP IV factors of cotton, *HOX1/2/3* (Supplementary Fig. 1a,b), are expressed in cotton fibre cells^14^,^15^. Southern blots showed double copies of *GhHOX3* in *G. hirsutum* and a single copy in the two models of the extant progenitor diploids, *G. herbaceum* (A_1_) and *G. raimondii* (D_5_; Supplementary Fig. 2), consistent with the sequenced *G. raimondii* genome^12^. The homoeologous GhHOX3-A and GhHOX3-D share 98% amino-acid sequence identity (Supplementary Fig. 1b). On the basis of single-nucleotide polymorphisms (SNPs) in the the A- and D-homoeologous sequences between *G. hirsutum* acc. TM-1 and *G. barbadense* cv. Hai7124 (ref. 16), *GhHOX3* genes were mapped on the 12th homoeologous chromosome pairs, A12 (Chr. 12) and D12 (Chr. 26; Supplementary Fig. 3), whereas *GhHOX1* and *GhHOX2* were localized to the fifth and the third homoeologous sets, respectively. By integrating with previously anchored quantitative trait loci within 20 cM, both *GhHOX3-A* and *GhHOX3-D* were associated with the quantitative trait loci for fibre length, uniformity and fineness (Supplementary Table 1). We subsequently surveyed 281 Chinese cultivars for SNP variation in *GhHOX3-A* (A12) and detected two SNPs, at positions 2560 (C/T) and 2761 (G/A). Association analysis showed that these two SNPs were significantly correlated with fibre length and uniformity in nine environments (Supplementary Table 2).

### GhHOX3 regulates cotton fibre elongation

**Figure 1:**
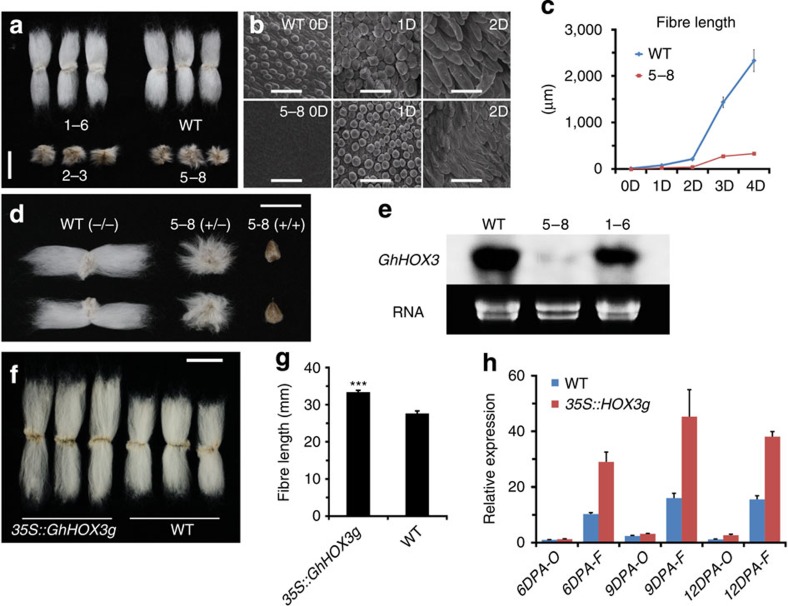
*GhHOX3* expression affects cotton fibre elongation. (**a**–**e**) Phenotypic analysis of *35S::GhHOX3* transgenic plants. Wild-type (WT) cotton and three transgenic lines are shown, of which two (5–8 and 2–3) exhibited co-suppression. (**a**) Images of fibre of *35S::GhHOX3* lines, T_2_ generation. Scale bar, 1 cm. (**b**) Scanning electron microscope (SEM) images of the ovule (0–2 DPA) of the co-suppression line 5–8 and the WT. Scale bars, 50 μm. (**c**) Mean fibre length at 0–4 DPA (mean±s.e.m., *n*>100). (**d**) Fibre of homozygous and heterozygous *35S::GhHOX3* co-suppression line 5–8 (T_6_ generation). Heterozygotes were generated by back-crossing the T_5_ plants to the WT. Scale bar, 1 cm. (**e**) Northern blot of *GhHOX3* expression in fibre (9 DPA) of the WT and the *35S::GhHOX3* (5–8 and 1–6) lines. (**f**) Images of fibre from a T_2_ plant of the *35S::GhHOX3g* (genomic) transgenic line. Scale bar, 1 cm. (**g**) Mature fibre length of a *35S::GhHOX3g* cotton line (mean±s.e.m., *n*=30, ***P<0.001, Student’s *t*-test). (**h**) Quantitative reverse transcription-PCR analysis of *GhHOX3* expression levels in *35S::GhHOX3g* ovule (O) and fibre (F), T_2_; DPA or D, DPA. Data are shown as mean±s.e.m. (*n*=3).

Designed to overexpress *GhHOX* genes in cotton, we used the *35S* promoter to drive the cDNA and transferred the constructs into *G. hirsutum*. While the *35S::GhHOX1* and *35S::GhHOX2* plants did not exhibit clear phenotypic effects, the *35S::GhHOX3* plants showed a dramatic decrease of fibre length (Supplementary Table 3), which was more pronounced in homozygous lines of T_2_ (Fig. 1a) and subsequent generations (>80% decrease). Cotton cultivars produce two types of fibre: the longer spinnable ‘lint’, and the shorter non-spinnable ‘fuzz’. Fibres of the homozygous transgenic lines were so short that the lint completely disappeared. Observation of 0–3 days post anthesis (DPA) ovules from a T_2_ line (5–8) revealed that fibre elongation was severely retarded, whereas initiation (density) was less affected (Fig. 1b,c). Other aspects of the transgenic plants appeared normal, except for the impaired trichome development on stem and leaf veins (Supplementary Fig. 4a,b). Genetic tests showed that the transgene exhibited a semi-dominant effect in the presence of the wild-type allele (Fig. 1d). The *GhHOX3* transcript level was sharply decreased in *35S::GhHOX3* transgenics (line 2–3 and 5–8), suggesting transgene co-suppression^17^. However, the expression levels of other *HOX* genes tested, such as *GhHOX1* and *GhHOX2*, were not affected (Fig. 1e; Supplementary Fig. 5a–c).

To confirm the co-suppression results, we downregulated the three *HOX* genes, respectively, by RNA interference. Again, only the *GhHOX3* RNA interference cotton plants produced shortened fibre, and the phenotype was indistinguishable from that of co-suppression (Supplementary Fig. 6a–e). To overcome co-suppression by cDNA, we used a genomic fragment to overexpress *GhHOX3* (*GhHOX3-A*) in cotton. Strikingly, compared with the wild-type, fibre length was increased up to 20% in the *35S::GhHOX3g* lines in which *GhHOX3* expression was elevated (Fig. 1f–h).

The cotton *HOX3* was expressed preferentially in rapidly elongating fibre at 3–9 DPA of both allotretraploid (*G. hirsutum*) and diploid (*G. arboreum*) cultivars grown in greenhouse (Supplementary Fig. 7a–c). Transcripts of both copies, namely *GhHOX3-A* and *GhHOX3-D*, were detectable in the fibre cells of *G. hirsutum*. When GhHOX3-A/D were fused with fluorescent proteins and transiently expressed in leaves, the signal was localized to the nucleus, consistent with their role as transcription factors (Supplementary Fig. 8).

### Two cell wall proteins are direct targets of *GhHOX3*

To identify the genes regulated by GhHOX3, digital gene expression tag profiling was performed to compare the transcripts of developing cotton fibre cells of the wild-type and the co-suppression lines, which identified over 300 differentially expressed genes, of which 78 were downregulated in co-suppression lines compared with the wild-type, under a strict screening criteria (Supplementary Data 1 and 2). Among them, the majority were classified into biological processes of cell growth, including cell wall formation, transcriptional regulation, signal transduction and metabolism. We then analysed the expression of these 78 genes in developing fibre cells by quantitative reverse transcription-PCR, which confirmed the downregulation of 76 in the co-suppression and upregulation of 49 out of the 76 in the overexpression lines (Supplementary Fig. 9). In *Arabidopsis*, the HD-ZIP IV factors bind to the conserved *cis*-element, L1-box, of target genes^18^,^19^. Of the 49 genes selected, 18 contain at least one L1-box in their promoters (Supplementary Table 4), indicating that they could serve as targets of GhHOX3.

**Figure 2:**
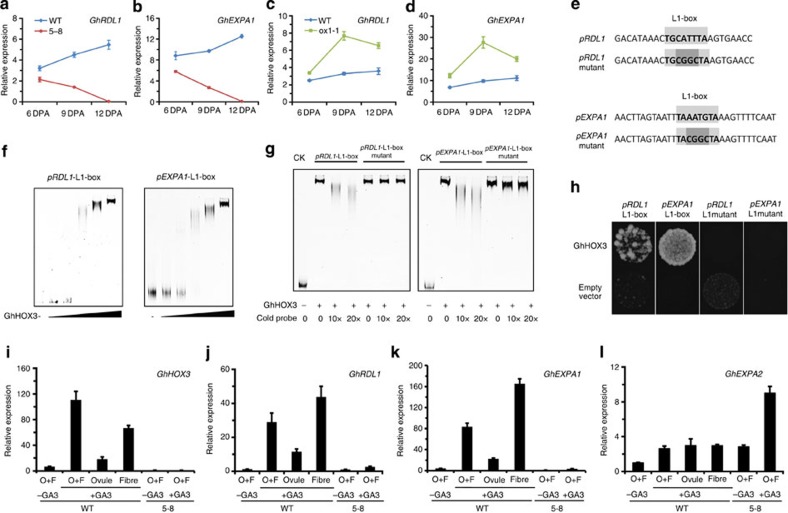
*GhRDL1* and *GhEXPA1* are direct targets of GhHOX3. (**a**–**d**) Quantitative reverse transcription-PCR analysis of *GhRDL1* and *GhEXPA1* transcripts in cotton fibre of the WT, co-suppression (5–8) and overexpression (ox1-1) plants. (**e**–**h**) GhHOX3 directly binds to *GhRDL1* and *GhEXPA1* promoters. Data are shown as mean±s.e.m. (*n*=3). (**e**–**g**) EMSA of GhHOX3 binding to L1-box from the *GhRDL1* and *GhEXPA1* promoters. The 6 × fragments of *GhRDL1* and *GhEXPA1* promoters containing the intact (upper) or the mutated (lower) L1-box (**e**) were incubated with gradient concentrations of maltose-binding protein (MBP)-GhHOX3 fusion protein (**f**). Labelled *GhRDL1* and *GhEXPA1* were incubated with MBP-GhHOX3 to compete with different concentrations of cold probes of intact or mutated L1-box (**g**). (**h**) Yeast one-hybrid assay of protein–DNA interaction, the 6 × fragments described in **e** were used. (**i**–**l**) Expression levels of *GhHOX3* and two downstream genes in ovule (O) and/or fibre (F), which were taken from *GhHOX3*-silenced line 5–8 and the WT cotton at 2 DPA and cultured *in vitro* with addition of the hormone GA_3_ (1 μM) for 6 days. *GhEXPA2*, expressed at a nearly equal level in ovule and fibre, was analysed as a control of GA treatments. Data are shown as mean±s.e.m. (*n*=3).

Putative targets of GhHOX3 included two cell wall protein genes, *GhRDL1* and *GhEXPA1*. *GhRDL1* has been shown to interact with GhEXPA1, an α-expansin that functions in wall loosening and cell expansion^9^. We previously showed that the *GhRDL1* promoter contains an L1-box and could be activated by cotton *HOX3* in transgenic *Arabidopsis*^14^. Interestingly, the *GhEXPA1* promoter also contains an L1-box. We found that the expression levels of both *GhRDL1* and *GhEXPA1* followed the change of *GhHOX3* expression: significantly downregulated in *GhHOX3*-silenced but upregulated in overexpressed lines (Fig. 2a–d). Electrophoretic mobility shift assays (EMSA) showed that GhHOX3 bound to the intact but not the mutated L1-box, from both promoters (Fig. 2e–g). This result was confirmed by yeast one-hybrid assay (Fig. 2h).

### Regulation of fibre elongation by GA involves GhHOX3

Phytohormones, including auxin, GA and ethylene, are important regulators of cotton fibre development^10^,^20^,^21^. As auxin and GAs are commonly involved in cell differentiation and growth, we tested if GhHOX3 functions downstream of a hormone. We cultured the cotton ovules (2 DPA) *in vitro* for 6 days, and found that GA_3_ promoted fibre elongation of the wild-type ovules in a dose-dependent manner, but not the *GhHOX3* co-suppression ovules (Supplementary Fig. 10). Indeed, GA_3_ treatments significantly upregulated *GhHOX3* and the downstream genes *GhRDL1* and *GhEXPA1* in the wild-type ovules, but their expression levels remained low in the co-suppression ovules (Fig. 2i–l). Increasing concentrations of auxin, however, did not exert an obvious effect on fibre elongation in our culture conditions, nor effect on the expression of *GhRDL1*, *GhEXPA1* and *GhHOX3* regardless of the cotton lines cultured (Supplementary Fig. 11a–d). Thus, GA plays an important role in regulating fibre elongation and GhHOX3 is required for this regulation.

**Figure 3:**
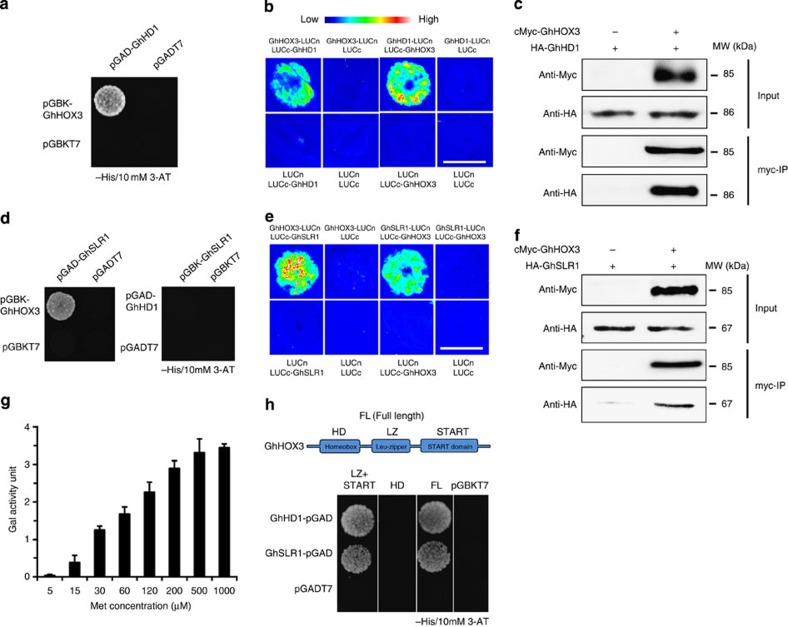
The DELLA protein GhSLR1 binds to GhHOX3 and interferes with the GhHOX3–GhHD1 interaction. (**a**) Yeast two-hybrid assay. pGAD-GhHD1 combined with pGBK-GhHOX3 conferred yeast growth on SD/-Leu/-Trp/-His plates supplemented with 10 mM 3-amino-1,2,4-triazole (3-AT). (**b**) BiFc assay. GhHOX3 and GhHD1 were interchangeably fused to the carboxyl- and amino-terminal of firefly luciferase (LUC, LUCc and LUCn), transiently co-expressed, and LUCc or LUCn was co-expressed with each other or with each un-fused target protein as the control. Fluorescence signal intensities represent their binding activities. Top bar, heat map’s scale of the signal intensity. GhHOX3 interacted with GhHD1. Scale bar, 1 cm in **b.** (**c**) Coimmunoprecipition (CoIP) of transiently co-expressed cMyc-GhHOX3 and HA-GhHD1 in leaves of *Nicotiana benthamiana*. Soluble protein extracts before (input) and after (IP) immunoprecipitation with anti-cMyc antibody-conjugated beads were detected by immunoblot with anti-HA antibody. (**d**) Yeast two-hybrid assay. GhHOX3, but not GhHD1, bound to GhSLR1 at 10 mM 3-AT. (**e**,**f**) *In vivo* BiFc (**e**) and CoIP (**f**) assays. GhHOX3 interacted with GhSLR1. Scale bar, 1 cm in **e**. (**g**) Yeast three-hybrid assay showing the influence of GhSLR1 on GhHOX3–GhHD1 binding represented by β-galactosidase activity, and the GhSLR1 expression was suppressed by increasing Met concentrations (data are shown as mean±s.e.m., *n*=3). (**h**) Domain deletion assay. Top, GhHOX3 contains three conserved domains. Below, yeast two-hybrid detection. GhHOX3 fragment containing both the Leu-zipper (LZ) and the START domains interacted with both GhHD1 and GhSLR1 in yeast, whereas the GhHOX3 homeodomain (HD) did not.

To further address how GhHOX3 functions, we screened a cotton fibre cDNA library for its interacting proteins. Two-thirds of the proteins identified were putative transcription factors potentially involved in phytohormone function and plant development (Supplementary Table 5). These included GhHD1, another HD-ZIP IV subfamily protein, which was reported to have a role in trichome and only a mild effect on fibre cell development^22^, and GhSLR1, a DELLA protein of the GA signalling component^23^. The expression of *GhHD1*, like that of *GhHOX1* and *GhHOX2*, was not affected in *GhHOX3* co-suppression lines (Supplementary Fig. 12), suggesting again that the gene silencing was specific to *GhHOX3* and the suppressed *GhHOX3* expression was responsible for the shorter fibre phenotype observed. In further yeast two-hybrid assays, GhHOX3 strongly interacted with GhHD1 (Fig. 3a), and the interaction was also observed in the biomolecular fluorescence complementation (BiFc) analysis (Fig. 3b) and the coimmunoprecipitation (CoIP) assay (Fig. 3c, Supplementary Fig. 17). DELLA proteins, which lack a DNA-binding domain, negatively regulate GA signalling by repressing activities of transcription factors through protein–protein interaction^24^. GhSLR1 is a functional orthologue of the *Arabidopsis* DELLA AtGAI, and widely expressed in various organs of cotton^25^ (Supplementary Fig. 13). The interaction between GhHOX3 and GhSLR1 was confirmed by yeast two-hybrid (Fig. 3d), BiFc (Fig. 3e) and CoIP assays (Fig. 3f, Supplementary Fig. 17). Interestingly, GhHD1 did not bind to GhSLR1 in yeast (Fig. 3d). We then tested if the cotton DELLA would compete with GhHD1 for binding to GhHOX3. Indeed, in the yeast three-hybrid assay, the GhHOX3–GhHD1 interaction was gradually weakened by increasing concentrations of GhSLR1 (Fig. 3g). Domain deletions revealed that both GhHD1 and GhSLR1 interacted with the GhHOX3 fragment containing both Leu-zipper and START domains, whereas the homeodomain was dispensable (Fig. 3h).

**Figure 4:**
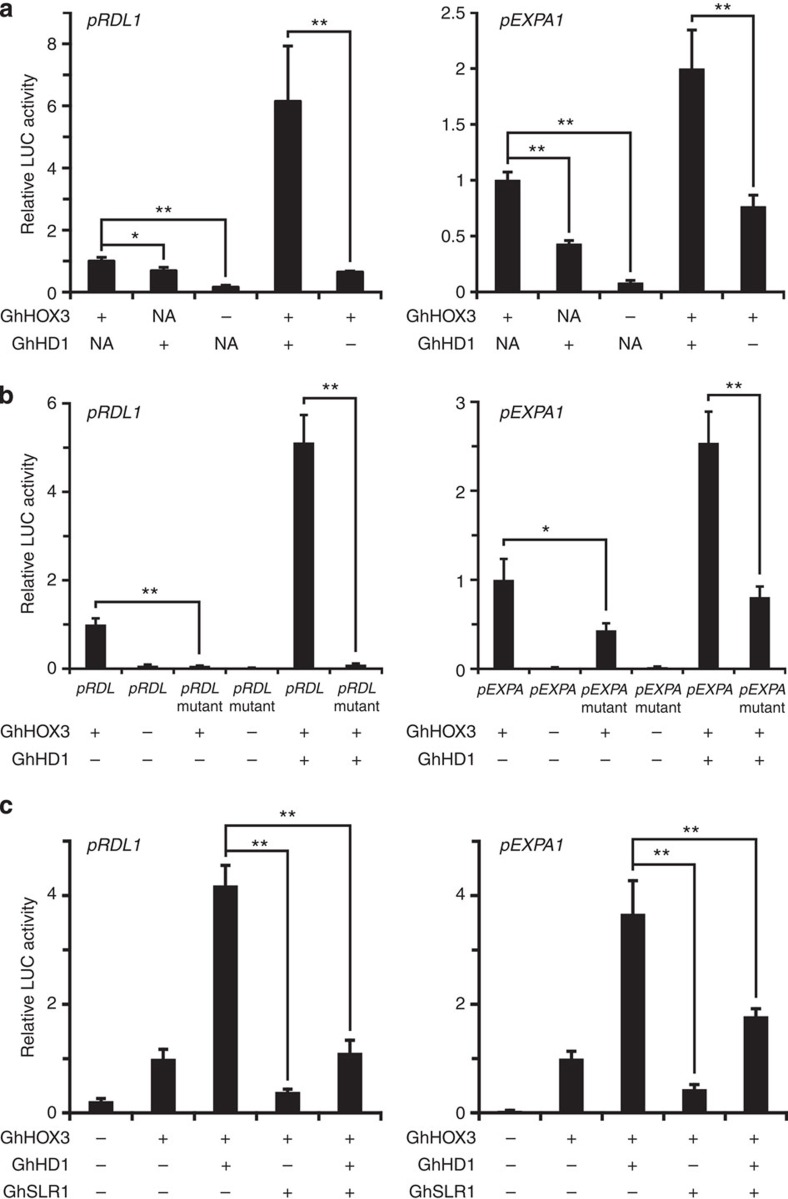
Transcriptional regulation of target genes by GhHOX3 and the effects of GhHD1 and GhSLR1. *GhRDL1* and *GhEXPA1* promoters were fused to the LUC reporter, respectively, and the promoter activities were determined by a transient dual-LUC assay in *Nicotiana benthamiana*. The relative LUC activities were normalized to the reference Renilla (REN) luciferase. The corresponding effector (+), empty vector (−) or neither (NA) were co-filtrated (data are presented as mean±s.e.m., *n*=3, *P<0.05; **P<0.01, Student’s *t*-test). (**a**) Effects of GhHOX3 and GhHD1 on activities of *RDL1* and *EXPA1* promoters. (**b**) Effects of L1-box mutation on the activity of *RDL1* and *EXPA1* promoters. The L1-box was mutated (mutant) as shown (Fig. 2e). (**c**) Inhibitive effects of GhSLR1 on activation of *RDL1* and *EXPA1* promoters by GhHOX3 and GhHOX3+GhHD1.

Given the fact that GA promotes cotton fibre elongation (Supplementary Fig. 10), we asked if the triple protein interactions would affect the transcriptional activation of target genes. A dual-luciferase assay system was employed for this purpose. The level of the luciferase activity controlled by *GhRDL1* and *GhEXPA1* promoters was elevated when GhHOX3 was expressed (Fig. 4a), but this activation was impaired when the L1-box was mutated (Fig. 4b). GhHD1 also activated the two promoters, but to a lesser extent than GhHOX3 (Fig. 4a). The *GhRDL1* L1-box conferred a higher degree of regulation, possibly due to its closer proximity to the coding region (Supplementary Table 4). Activation of GhHOX3 to *GhRDL1* or *GhEXPA1* promoters was significantly enhanced by GhHD1 (Fig. 4a), and this enhancement was synergistic as the combinatorial effect was more than additive (particularly to *GhRDL1*), and the amounts of agrobacterial cells infiltrated into leaves in each experiment, as well as the amounts of the proteins expressed, were comparable (Supplementary Figs 14,17). In contrast, the promoter activation, either by GhHOX3 alone or by the two HD-ZIP IV proteins in combination, was substantially repressed by GhSLR1 (Fig. 4c). These results were further supported by EMSA assays, in which GhHD1 or GhHOX3 alone, as well as their combination, bound to the L1-box from both *GhRDL1* and *GhEXPA1* promoters, and such an interaction was indeed disturbed by application of GhSLR1 in the system (Supplementary Fig. 15).

## Discussion

**Figure 5:**
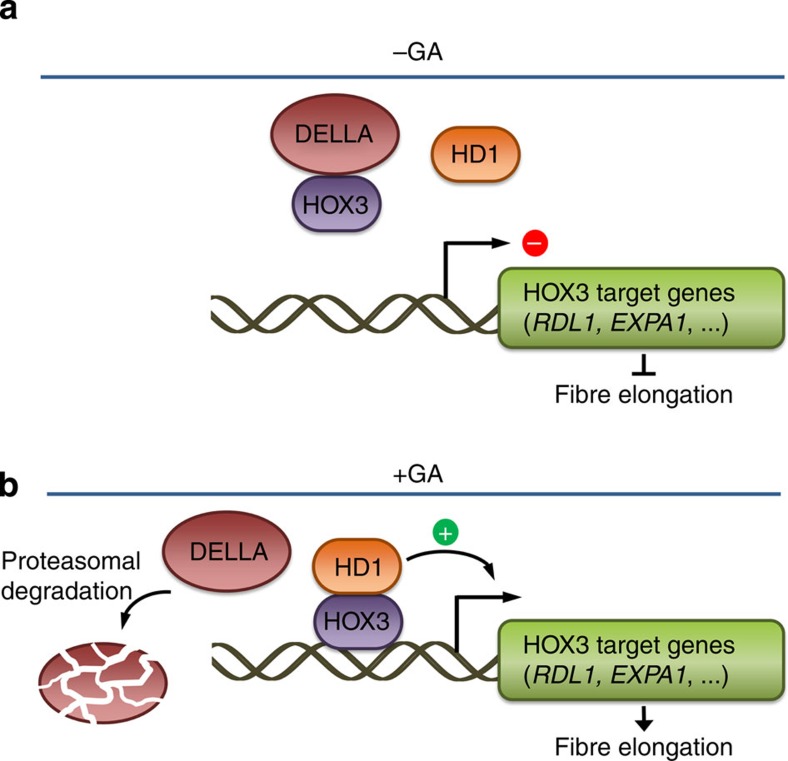
A model for the regulation of cotton fibre elongation by GhHOX3 and the phytohormone GA. (**a**) In cotton fibres, DELLA proteins bind to HOX3 to prevent its binding to other HD-ZIP transcription factors, repressing their transcriptional activation to target genes. (**b**) GAs trigger degradation of DELLAs, releasing HOX3 protein to interact with other HD proteins, such as HD1, allowing the activation of target genes, including *RDL1* and *EXPA1*, to promote cotton fibre elongation.

We have shown that the interaction of GhHOX3 with GhHD1 results in a much higher activity of gene activation than either alone, and DELLA (GhSLR1) negatively affects their activity and integration. In plant cells, DELLAs are degraded in response to GA^26^. Our results uncover a new molecular mechanism underlying the role of GA in promoting cotton fibre elongation (Fig. 5).

PlantTFDB^27^ predicted 15 HD-ZIP IV subfamily transcription factors in the diploid cotton *G. raimondii* genome^12^. At least five of their orthologues are expressed in fibre cells of *G. hirsutum*^14^,^15^,^22^. Among the four HD-ZIP factors examined here, GhHOX1 is the most similar to, and could functionally substitute for, the trichome regulator AtGL2 in *Arabidopsis*^15^, whereas GhHOX3 belongs to a distant clade that contains AtHDG11/12 (Supplementary Fig. 16). Overexpression of *AtHDG11* in *Arabidopsis*, tobacco and rice conferred drought tolerance with extensive root architecture change and reduced leaf stomatal density^28^,^29^. However, our data identify GhHOX3 as a core regulator of fibre elongation, and other HD-ZIP IV proteins, such as GhHD1, could function as accessory factors in the regulatory complex in activating downstream genes of the fibre elongation pathway. Further characterization of the regulatory network centering on GhHOX3 could help to improve cotton fibre quality and yield by increasing fibre length through molecular breeding. Moreover, the direct interaction between DELLA and a specific HD-ZIP IV protein sheds new light on the biochemical mechanism of GA, a widely acknowledged green revolution hormone, in controlling cell elongation, plant height and architecture. Almost simultaneously, a very recent work demonstrated that, during *Arabidopsis* seed germination, DELLAs affect epidermal cell elongation by sequestering HD-ZIP IV transcription factors from activating downstream gene expression^30^. This result, together with our own, strongly suggests that such mechanism is highly conserved in plants.

## Methods

### Plant materials

Cotton plants (*Gossypium hirsutum* cv. R15, *G. arboreum* cv. Qinyangxiaozi, *G. herbaceum* and *G. raimondii*) were grown in a greenhouse at 28±2 °C under a 14-h light photoperiod. Ovules were harvested at 0–18 DPA at a 3-day interval. Fibres were collected by scraping the ovule in liquid nitrogen. For genetic analysis, 281 cultivars or lines of *G. hirsutum* were grown in different areas of China: Akesu (northwestern region), Anyang (Yellow River basin) and Nanjing (Yangtze River basin), respectively, from 2007 to 2009. *Nicotiana benthamiana* was grown at 22±2 °C under a 16-h light photoperiod.

### Genetic analysis

The population comprising of 138 BC_1_ individuals, generated from the cross of (*G. hirsutum* acc. TM-1 × *G. barbadense* cv. Hai7124) × TM-01 (ref. 31), was used to map the *GhHOX3* gene. According to the different subgenomic sequences between TM-01 and Hai7124, SNP primers for *GhHOX3* in corresponding subgenomes were designed to produce the polymorphisms in the two parents. Separation of *GhHOX3* in the BC_1_ mapping population was then detected by polyacrylamide gel electrophoresis (PAGE). Join Map3.0 (ref. 32) was employed to perform the linkage analysis, and the result was integrated with a previously constructed genetic linkage map^16^. MapChart was used to complete the chromosomal localization. Sequences of all primers used in this investigation are listed (Supplementary Table 6). The A-sugenome gene (*GhHOX3-A*) was used in this investigation unless specifically indicated.

For EcoTILLING assays, young leaves from each cotton variety were freshly harvested for total genomic DNA extraction as described^33^. DNA from all samples was quantified using a spectrophotometer and normalized to a concentration of 20–60 ng μl^−1^. The nuclease CEL I was extracted from celery^34^, and enzymatic activity was tested according to the Surveyor Mutation Discovery kit (Transgenomics) protocol. For nucleotide polymorphism with EcoTILLING, gene- and subgenome-specific primers were designed based on the sequence of *GhHOX3* by a semi-nested PCR using Ex-Taq polymerase (TaKaRa). The semi-nested PCR for *GhHOX3-A* used the same forward primer and two different reverse primers. For SNP screens^35^, each of the semi-nested PCR products was mixed with that of TM-1. Heteroduplexes would form in the mixture of each two PCR products if SNPs existed between the variety and TM-1. The heteroduplexes could be digested by CEL I. After cleavage, the heteroduplexes were visualized using PAGE. The cut DNAs were visible as bands and those with faster mobility than the full-length product were considered a polymorphism. Once a polymorphism was identified, the corresponding DNA sample was amplified using gene- and subgenome-specific primers. The resulting PCR fragment was sequenced, and each polymorphic site was sequenced from more than one accession to confirm that only two alleles segregated at any specific site.

### Cotton transformation

For the *p35S::GhHOX3* construct, the open reading frame (ORF) of *GhHOX3* was PCR amplified from the *G. hirsutum* cv. R15 fibre cDNA library using PrimeSTAR HS DNA polymerase (TaKaRa) and cloned into the *pCAMBIA2301/35S* vector (CAMBIA). For *35S::GhHOX3g*, the coding region of *GhHOX3-A* amplified from genomic DNA was cloned into the *pGWB5* vector (Invitrogen). For *35S::dsHOX3*, sense and antisense *GhHOX3* fragments separated by a 120-bp intron of *A. thaliana RTM1* gene^36^,^37^ were cloned into *pBI121/35S* (Clontech).

Binary constructs were introduced into *Agrobacterium tumefaciens* strain LBA4404. The *Agrobacterium*-mediated cotton transformation was performed using hypocotyl segments of *G. hirsutum* cv. R15 as explants, as described^38^. After callus induction, proliferation, embryogenic callus induction, embryo differentiation and finally plantlet regeneration, the plantlets were transferred into pots and grown in the greenhouse.

### Nucleic acid and expression analysis

Genomic DNA of cotton was isolated with CTAB (cetyltrimethyl ammonium bromide) extraction solution (2% CTAB, 0.1 M Tris, 20 mM EDTA, 1.4M NaCl, pH=9.5) (ref. 39). For Southern blot analysis, total genomic DNA samples were digested by restriction enzymes (5U enzyme per 1 μg DNA) overnight, separated on 1.0% agarose gels (20 μg for each lane) and transferred to Biodyne B membranes (Pall). The fragment of *GhHOX3* or the *NPTII* gene amplified from pCAMBIA2301 (CAMBIA) was used as a probe, which was labelled with ^32^P-dCTP using a Random Primer DNA Labeling kit (TaKaRa). Membranes were hybridized and washed according to a standard protocol.

Cotton RNA was extracted also with the 1% cetyltrimethyl ammonium bromide (CTAB) solution, precipitated by 2 M LiCl^40^. For Northern blots, total RNAs (10 μg each lane) were resolved on 1.2% formaldehyde agarose gels and transferred onto Hybond-N^+^ nylon membranes (GE Healthcare-Amersham). Hybridization with the ^32^P-labelled probe and membrane washing were performed following standard protocols. Total RNAs of 1 μg, after treatment with DNase I, were used for cDNA synthesis with oligo(dT) primers and M-MLV (Moloney Murine Leukaemia Virus) Reverse Transcriptase (Invitrogen). Quantitative real-time reverse transcription-PCR was performed with SYBR-Green PCR Mastermix (TaKaRa), and amplification was real-time monitored on a cycler (Mastercycler RealPlex, Eppendorf). The *G. hirsutum histone-3* gene (*GhHIS3*) was used as an internal reference.

### Digital gene expression analysis

Total RNA of 6-DPA fibres from the wild-type (*G. hirsutum* cv. R15) and *GhHOX3* co-suppression line 5–8 was extracted with the the CTAB solution, and the mRNA was separated using oligo-dT magnetic beads, and sheared into short fragments (≈200 bp) in the fragmentation buffer. First-strand cDNA was synthesized by random hexamer primers (mRNA fragments as templates). The double-stranded cDNA was synthesized and purified with a QiaQuick PCR extraction kit (Qiagen), and washed with elution buffer for end repair and single-nucleotide (adenine) addition, followed by the ligation of sequencing adaptors. The fragments were purified by agarose gel electrophoresis and sequenced with a high-throughput sequencer (HiSeq 2000, Illumina) with a read length of 50 bp. Three biological replicates were performed separately.

After filtration, cleaned reads were mapped to the *G. raimondii* genome^12^ using SOAPaligner (SOAP2 (ref. 41), BGI). For annotation, genes mapped were used as query sequences to search against the non-redundant protein database of NCBI, and the Kyoto Encyclopedia of Genes and Genome (KEGG) pathways database. Gene expression levels were normalized and calculated as reads per kb per million reads values^42^. Significance of differential gene expression was determined (false discovery rate ≤0.001, absolute value of log_2_ ratio ≥1) by random test (*P*<0.05)^43^.

### Ovule culture

The 2-DPA cotton bolls were harvested and sterilized with 0.1% (w/v) HgCl_2_ solution for 15 min and washed three times with sterile distilled water. The sterilized ovules were taken out and placed in liquid BT media, supplemented with different concentrations of auxin (indole-3-acetic acid, IAA, Sigma) and GA (Gibberellic acid, GA_3_, Sigma), and cultured in the dark at 30 °C for 6 days^21^ (Supplementary Figs 10 and 11). Ovules, harvested at 2 DPA, were cultured for 6 days before subsequent analysis.

### Yeast assay for protein–protein and protein–DNA interactions

A cDNA library was constructed from mRNAs of the 6-DPA fibres using the CloneMiner II cDNA Library Construction Kit (Invitrogen). The cDNA library was cloned into *pDEST22* and *GhHOX3* cDNA was inserted into *pDEST32*. A yeast library screen was performed using *GhHOX3-pDEST32* according to the manufacturer’s manual (Invitrogen).

Yeast one-hybrid, two-hybrid and three-hybrid analyses were performed using the Matchmaker GAL4 Two-Hybrid System according to the manufacturer’s manual (Clontech). Plasmids were transferred into yeast strain AH109 by the LiCl-PEG method.

For the yeast one-hybrid assay, the 6 × promoter segments of *GhRDL1* and *GhEXPA1* (intact or mutated L1-box, generated by Generay) were inserted into *pHIS2.1* (Clontech), and assayed following the manufacturer’s manual. Yeast two-hybrid assays were performed with the full-length or truncated ORFs of *GhHOX3* inserted into *pGBKT7* (Clontech) and *GhHD1* or *GhSLR1* into *pGADT7* (Clontech). Plasmids were co-transferred into yeast, and the interactions were detected on SD/-Leu/-Trp/-His selective plates containing 10 mM 3-AT (3-amino-1,2,4,-triazole). Three independent clones for each transformation were tested. Empty vectors of *pGADT7* and *pGBKT7* were used as controls. For the yeast three-hybrid assay, *GhHD1* and *GhSLR1* were inserted into *pBridge* (Clontech), forming a *GhHD1-GhSLR1/pBridge* construct, and *GhHOX3* was placed in *pGADT7*. Plasmids were co-transferred into yeast and plated on SD-Leu-Trp selective dropout medium. Colonies were transferred to the appropriate SD/-Leu/-Trp/-His selective dropout liquid medium with different concentrations of methionine (Met). *GhSLR1* expression from the *pBridge* construct was controlled by the *pMet25* promoter, and the GhSLR1 level was increased along with the decreasing concentrations of Met. Specific activities of β-galactosidase were detected according to the manufacturer’s manual.

### BiFc assay

BiFC assays were performed as previously reported^44^,^45^. For constructs, ORFs of *GhHOX3*, *GhHD1* and *GhSLR1* were PCR amplified and cloned into JW771 and JW772 vectors^44^, respectively. Each ORF was fused to the carboxyl-terminal half (cLUC-GhHOX3/GhHD1/GhSLR1) and the amino-terminal half (GhHOX3/GhHD1/GhSLR1-nLUC) of luciferase (LUC), respectively; cLUC and nLUC were used alone as controls. *Agrobacterium* cells were resuspended in infiltration buffer (10 mM MgCl_2_, 10 mM MES (2-(*N*-morpholino)ethanesulfonic acid) pH5.7, 150 μM acetosyringone) at OD_600_=0.8. *35S::P19-HA* and the suspension were co-infiltrated to inhibit gene silencing^46^. After a 3-day culture, a total of 0.8 mM luciferin was infiltrated into the abaxial side of *N. benthamiana* leaves and the LUC activity was monitored. The following pairs of constructs were used for co-infiltration: cLUC-GhHOX3 and GhHD1-nLUC, cLUC-GhHD1 and GhHOX3-nLUC, cLUC-GhHOX3 and GhSLR1-nLUC and cLUC-GhSLR1 and nLUC-GhHOX3, together with their respective controls.

### CoIP assay

The soluble proteins were extracted using a extraction buffer (pH 7.5) containing 100 mM of Tris-HCl, 5 mM EDTA (ethylene diamine tetraacetic acid), 100 mM NaCl, 0.2% Nonidet P-40, 1.0% Triton-X-100, 1 mM DTT (dithiothreitol), 1 mM PMSF (phenylmethanesulfonyl fluoride), 100 mM MG-132 (Sigma-Aldrich) and protease inhibitor cocktail (Roche). Immunoprecipitation was performed with anti-cMyc-affinity beads (Sigma-Aldrich). Lysates were incubated with the prewashed beads for 1 h at 4 °C. The beads were then washed three times and solubilized in an appropriate volume of extraction buffer with 5 × SDS loading buffer (Tiangen). GhHOX3-3 × HA and cMyc-GhHD1/cMyc-GhSLR1 fusion proteins were detected by immunoblot with 1:1,000 diluted anti-HA antibody (Roche) and 1:1,500 diluted anti-Myc antibody (Millipore), respectively.

### Protein immunoblot (western blot) analysis

Proteins were separated with 12% SDS-PAGE and transferred to a polyvinylidene fluoride membrane (GE Amersham). Blots were blocked for 1 h in PBS, with 5% skimmed milk powder (Oxoid) and 0.1% Tween 20 (Sigma-Aldrich), and incubated with corresponding antibody in blocking buffer for 1 h at room temperature. GhHOX3 monoclonal antibody was raised against the residues 1–200 of GhHOX3 protein (Abmart). After incubation, the blots were washed three times and incubated with 1:10,000 diluted anti-mouse HRP (horseradish peroxidase)-conjugated secondary antibody (Abmart) for 1 h at room temperature. After washing thrice, the HRP was detected using SuperSignal West Femto Maximum Sensitivity Substrate (Thermo) following the manufacturer’s protocol.

### EMSA

The ORF of *GhHOX3* and *GhHD1*, in frame, was fused to the maltose-binding protein tag of the expression vector *pMAL-C2* (New England Biolabs), and the recombinant proteins were affinity purified following the manufacturer’s manual. The ORF of *GhSLR1* and the yellow fluorescence protein gene were in frame fused to the glutathione *S*-transferase tag of the expression vector *pGEX-4T-1* (GE Healthcare), respectively, and the recombinant proteins were affinity-purified using Glutathione Sepharose 4B (GE Healthcare) following the manufacturer’s manual. The 6 × promoter segments of *GhRDL1* and *GhEXPA1*, containing the intact or mutated L1-box *cis*-element, were labelled with Cy5 on both ends. The assay was performed by incubation of the DNA fragment with the purified protein at 25 °C for 30 min, separated with 5% native PAGE in 0.5 × TBE (Tris/Borate/EDTA) buffer (10 V cm^−1^, 4 °C). Fluorescence was observed with an image scanner (FLA-9000, FUJIFILM).

### Dual-luciferase (Dual-LUC) assay

The assay was performed as reported^47^. Briefly, The *GhRDL1* and *GhEXPA1* promoters, with intact or mutated L1-boxes, were inserted into *pGreen-LUC*, respectively, to drive the firefly LUC reporter gene with the Renilla (REN) luciferase controlled by the constitutive 35S promoter on the same plasmid as a reference to normalize infection efficiency. The constructs were transferred into *Agrobacterium tumefaciens* (strain GV3101) with the helper plasmid, *pSoup-P19*, which encodes a repressor of co-suppression. The transformed *Agrobacterium* cells were mixed with the *Agrobacterium* strains harbouring the effectors or the empty vector control, in a volume ratio of 1:2.

Transient transformation was conducted by infiltration of the *Agrobacterium* mixtures into the abaxial side of *N. benthamiana* leaves using a syringe. After culturing for 3 days, the infected area was harvested for total protein extraction. The supernatant of total proteins was used with the Dual-Luciferase Reporter Assay System (Promega) following the manufacture’s manual, and the fluorescent values of LUC and REN were detected with a luminometer (BG-1, GEM Biomedical Inc.), successively. The value of LUC was normalized to that of REN. Three biological repeats were measured for each combination.

### Microscopic observation

Images were generated with an optical microscope (BX51, Olympus) and fibre length was measured with ImageJ (Wayne Rasband). For subcellular localization, *35S::GFP-GhHOX3-A/D* and *35S::GhHOX3-A/D-linker-venus* (yellow fluorescence protein) were constructed and transiently expressed in *N. benthamiana* leaves. After 3 days, the leaf tissues were observed under a laser scanning confocal microscope (LSM510, Zeiss). For scanning electron microscope images, cotton ovules (0–2 DPA) were attached with colloidal graphite to a copper stub, frozen under vacuum and visualized with a scanning electron microscope (JSM-6360LV, JEOL).

## Author contributions

C.-M.S., T.-Z.Z. and X.-Y.C. conceived and designed the research. C.-M.S., X.-X.S., B.Z., L.-M.C., X.-F.Z., X.-Y.G., C.-Q.Y., H.-Y.Z. and Y.-D.Z. performed the experiments. C.-M.S., C.-Q.Y., L.-J.W., Z.J.C., G.-J.H, J.F.W., W.-Z.G., B.-L.Z. and T.-Z.Z. contributed reagents, materials and/or data analysis. C.-M.S., Z.J.C., T.-Z.Z., J.F.W. and X.-Y.C. wrote the article.

## Additional information

**How to cite this article:** Shan, C.-M. *et al*. Control of cotton fibre elongation by a homeodomain transcription factor GhHOX3. *Nat. Commun.* 5:5519 doi: 10.1038/ncomms6519 (2014).

**Accession codes:** Sequence data for two homoeologous genes of *GhHOX3* have been deposited in GenBank/EMBL/DDBJ database under the accession codes KJ595847 (*GhHOX3-A*) and KJ595848 (*GhHOX3-D*).

## Supplementary Material

Supplementary Information Supplementary Figures 1-17, Supplementary Tables 1-6 and Supplementary References

Supplementary Data 1 Pairwise comparison of differentially expressed genes in 6-DPA fiber of *GhHOX3* co-suppression line 5-8 and the wild-type *G. hirsutum* cv. R15.

Supplementary Data 2 List of filtered differentially expressed genes in 6-DPA fiber of *GhHOX3* co-suppression line 5-8 compared to the wild-type *G. hirsutum* cv. R15.
