# Supplementary material for: Control of cotton fibre elongation by a homeodomain transcription factor GhHOX3
Source: Nat Commun. 2014 Nov 21;5:5519. doi: 10.1038/ncomms6519 (PMC4263147; doi:10.1038/ncomms6519)
Supplement: Supplementary Information — Supplementary Figures 1-17, Supplementary Tables 1-6 and Supplementary References [file ncomms6519-s1.pdf]

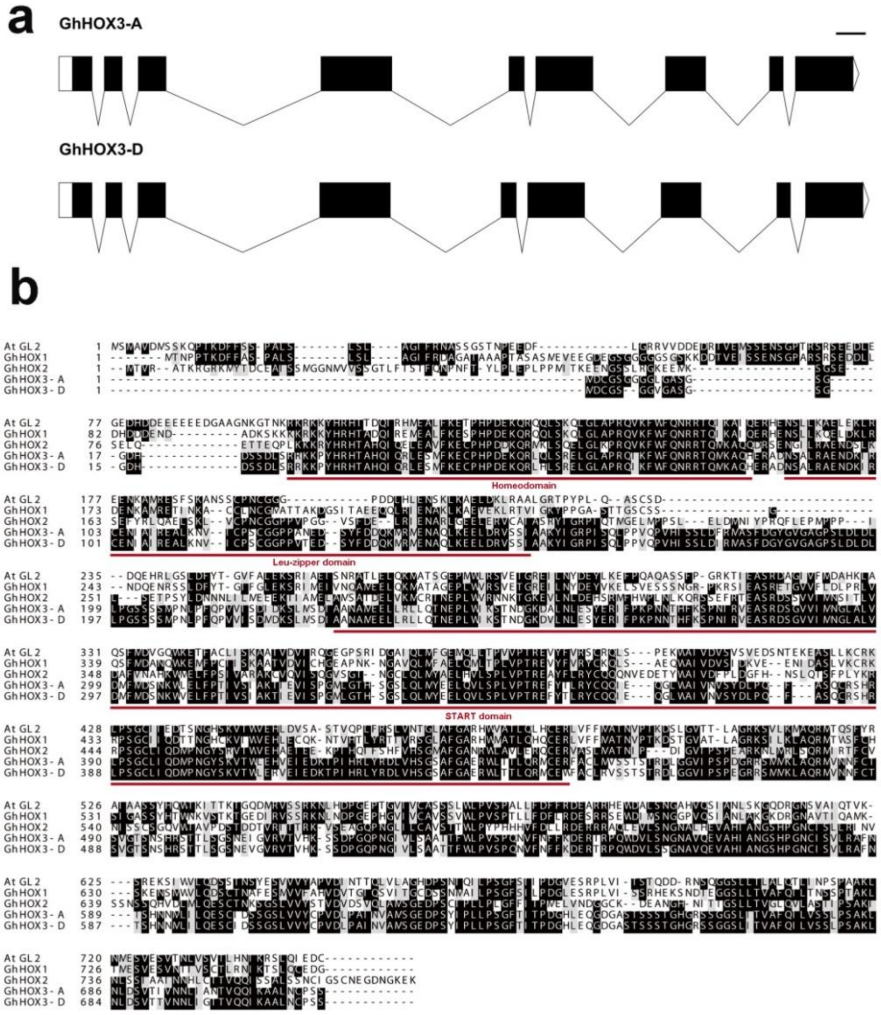

Supplementary Figure 1 Overview of *GhHOX3* gene.

(a) Schematic show of cotton (*Gossypium hirsutum*) *HOX3* gene, *GhHOX3*. The length of both copies of *GhHOX3* coding region is around 5.4 kb, containing 9 exons and 8 introns. Scale bar, 200bp. **(b)** Alignment of amino acid sequences of GhHOX1 (GenBank: AF530913), GhHOX2 (GenBank: AF530914), GhHOX3 and *Arabidopsis thaliana* GLABRA2 (AtGL2, GenBank: L32873). *GhHOX3-A* (GenBank: KJ595847) and *GhHOX3-D* (GenBank: KJ595848) sequences were obtained by amplifying *HOX3* cDNA from 6DPA fiber cells. All proteins aligned here belong to the homeodomain-leucine zipper (HD-ZIP) IV subfamily. The homoeologous GhHOX3-A and GhHOX3-D are 98% identical with amino acid sequences. Protein sequences were aligned by CLUSTALW (v1.81) following the default parameters. Conserved domains of

HD-ZIP IV proteins are underlined in red.

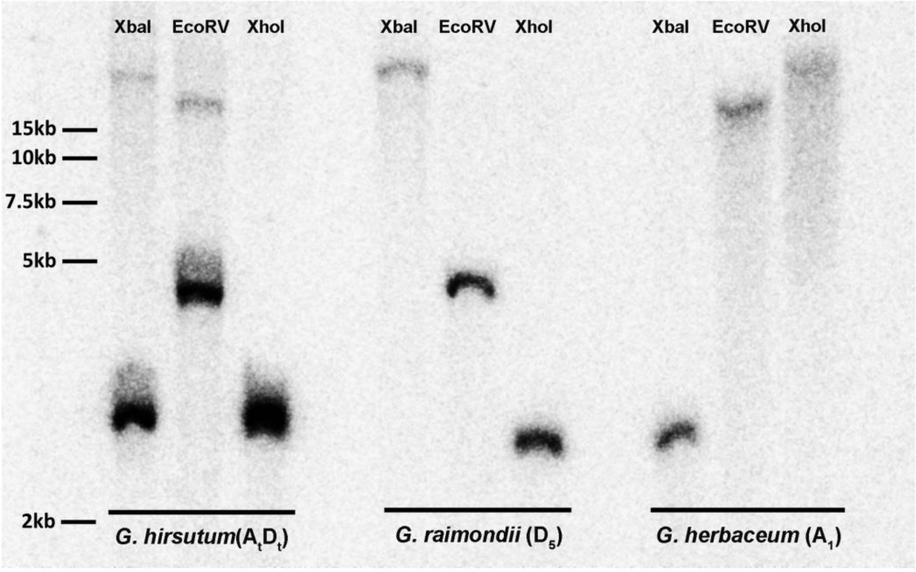

Supplementary Figure 2 Southern blot detection of *GhHOX3*.

Genomic DNAs from *G. hirsutum* cv. R15 (allotetraploid A_tD_t genome), and its two proposed extant progenitor diploid species, *G. herbaceum* (A_1 genome) and *G. raimondii* (D_5 genome), were digested by Xba I, EcoR V and Xho I, respectively, and then hybridized with a *GhHOX3*-specific probe.

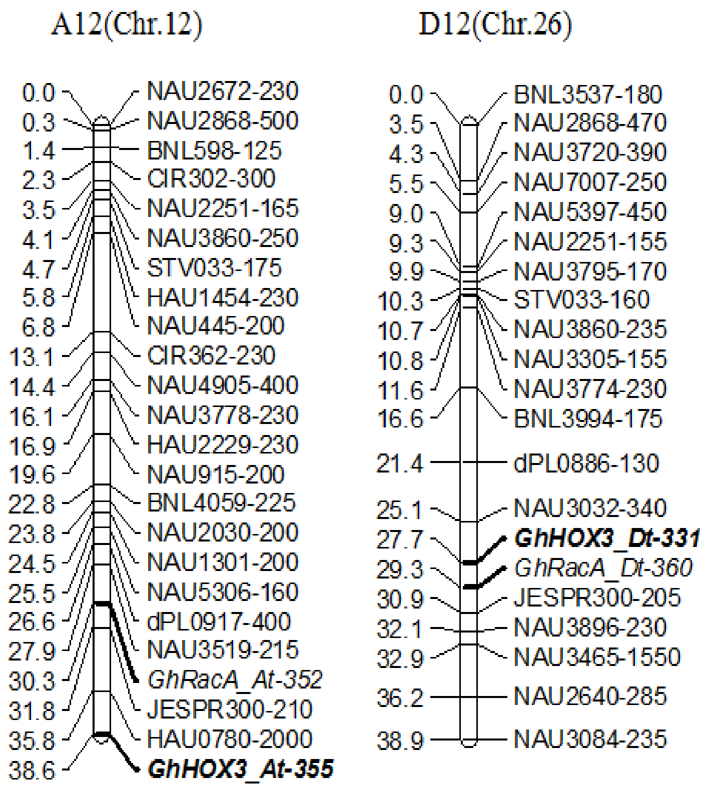

Supplementary Figure 3 Molecular mapping of *GhHOX3* genes. The genes were localized in the 12th homoeologous chromosome set of allotetraploid cotton, using a population generated from the cross of (*G. hirsutum* acc. TM-1×*G. barbadense* cv. Hai7124)×TM-1. See also **Online Methods.**

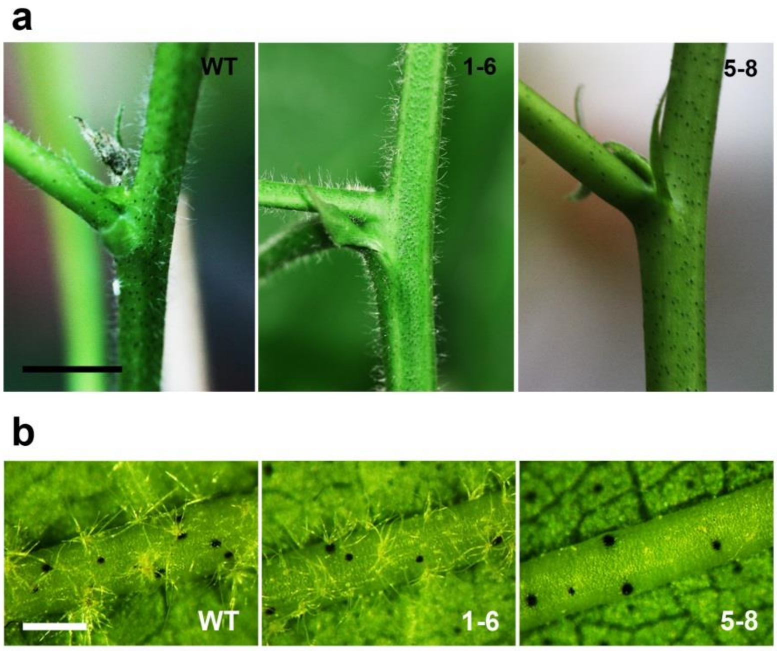

Supplementary Figure 4 Trichome phenotypes of T_2 plants of *35S::GhHOX3* transgenic lines 5-8 and 1-6.

(a-b) Images of trichome phenotypes on stem **(a)** and leaf vein **(b)**. *GhHOX3* expression was silenced in line 5-8 due to co-suppression, but not or only slightly affected in line 1-6 (see **Figure 1e**). The wild-type (WT) plant of *G. hirsutum* cv. R15 is shown as control. Scale bars, 1 cm in **(a)**, and 1 mm in **(b)**.

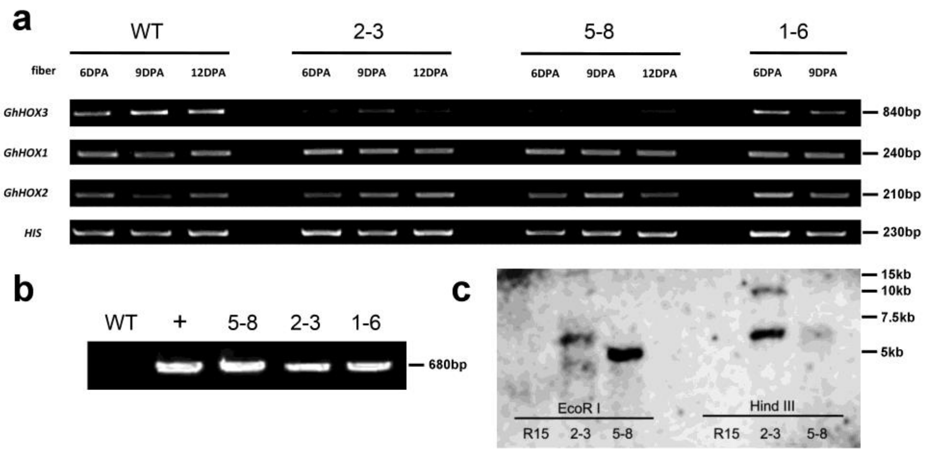

Supplementary Figure 5 Analysis of *35S::GhHOX3* transgenic cotton lines. **(a)** RT-PCR analysis of *GhHOX1/2/3* expression levels in fibers of T_2 plants of three *35S::GhHOX3* transgenic lines (2-3, 5-8, 1-6) and of the wild-type *G. hirsutum* cv. R15 (WT). Fiber cells of different developing stages as indicated were analyzed and *GhHIS3* was used as a reference. The analysis was repeated 3 times with consistent results. PCR cycles for GhHOX1/2/3 was 25, 25 and 28, respectively. **(b)** PCR and **(c)** Southern blot detection of the transgene in T_2 plants of transgenic lines. Genomic DNAs were digested with EcoR I and Hind III, and then hybridized with a *NPT II* probe.

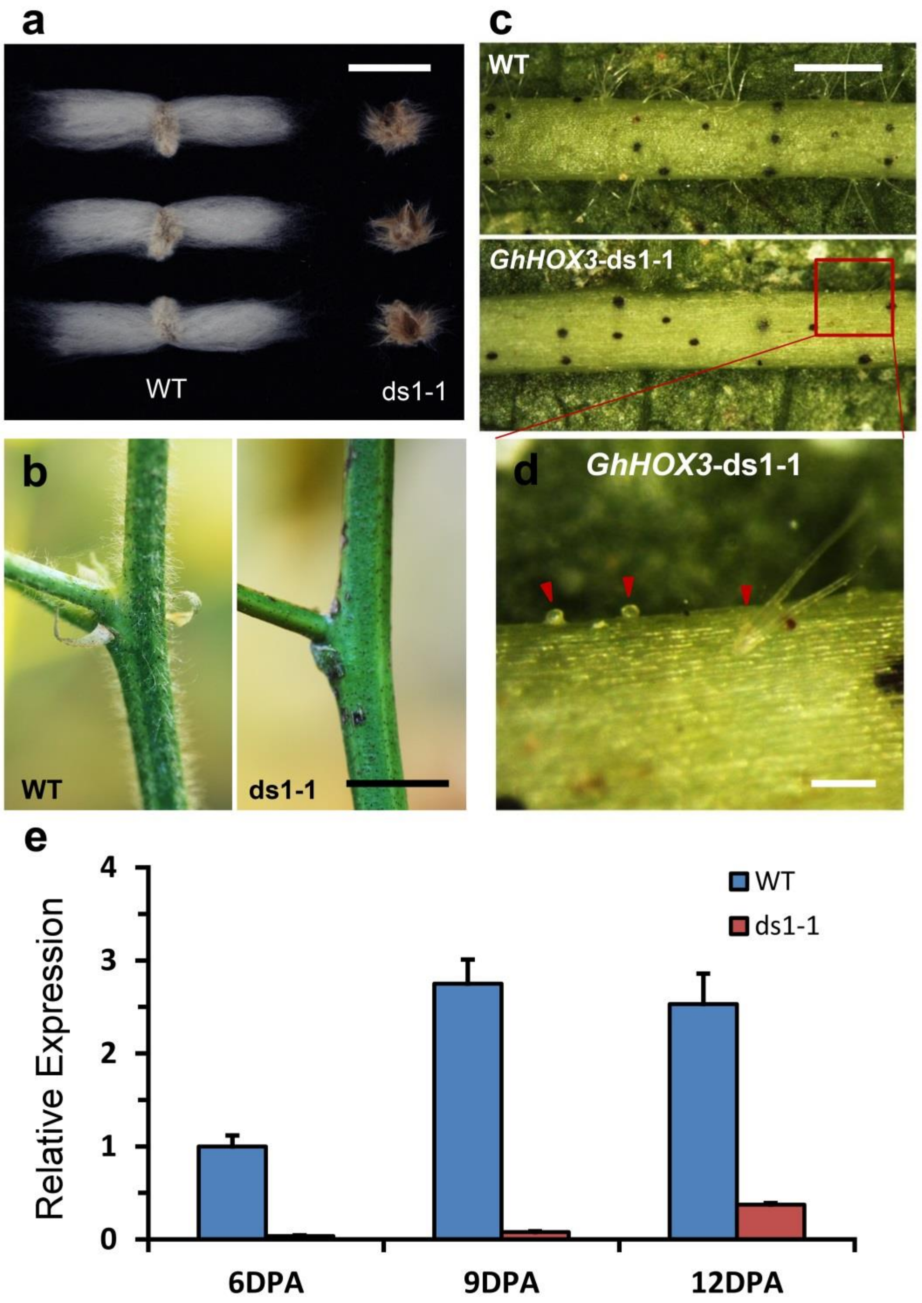

Supplementary Figure 6 Fibers and stem trichomes of *GhHOX3* dsRNA transgenic plants**.**

(a) Fiber of a *GhHOX3* dsRNA line, ds1-1. **(b-d)** Trichome on stem **(b)** and leaf vein **(c,d)** of the line ds1-1 and the wild-type (WT) cotton; underdeveloped trichome shown in magnified square **(d)** is indicated by red arrow. Scale bars, 2 cm in **(a)**, 1 cm in **(b)**, 1 mm in **(c)**, and 100 μm in **(d). (e)** Expression levels of *GhHOX3* in fibers of the wild-type and the RNAi line (ds1-1) examined by

qRT-PCR, data are shown as mean ± s.e.m. (n=3).

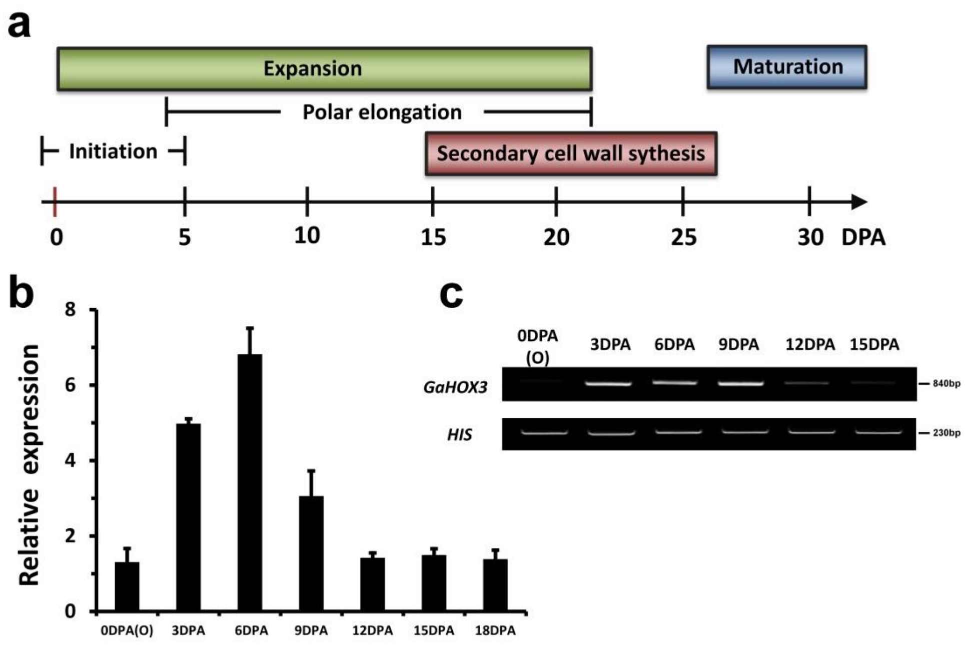

Supplementary Figure 7 *HOX3* expression in cotton fiber cells of allotetraploid (*G. hirsutum*) and diploid (*G. arboreum*) cultivars grown in green house.

(a) Sketch of cotton fiber developing stages. **(b,c)** *GhHOX3* expression in ovule (0 DPA) and fiber collected at different DPA as indicated. The expression in *G. hirsutum* was analyzed by qRT-PCR **(b)** and in *G. arboreum* by RT-PCR **(c)**. For qRT-PCR, data are shown as mean ± s.e.m. (n=3). For RT-PCR, analysis was repeated 3 times with consistent results.

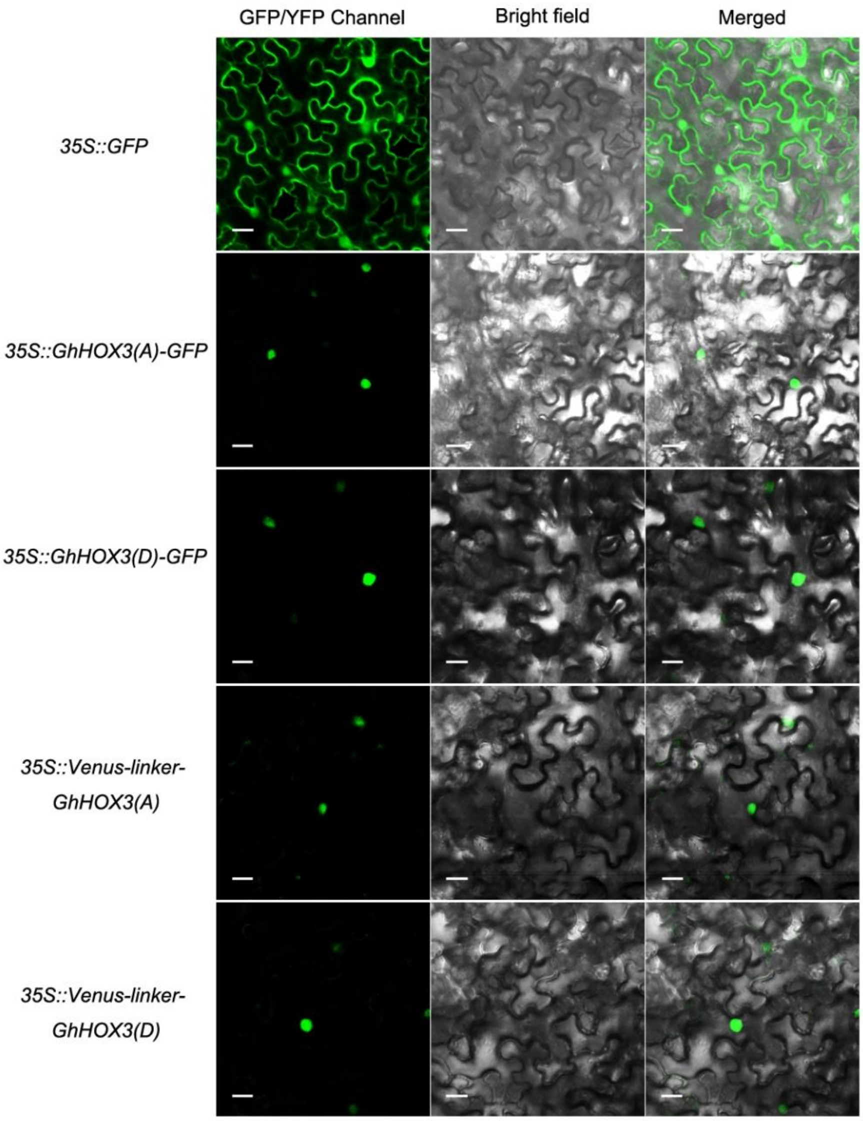

Supplementary Figure 8 Subcellular localization of GhHOX3.

GFP/Venus(YFP) fluorescence proteins were fused to the C-terminal or N-terminal of GhHOX3-A and GhHOX3-D, respectively, and transiently expressed in *N. benthamiana* leave cells via Agrobacteria infiltration. All the fluorescence signals were localized to the nucleus. Scale bars, 20 μm.

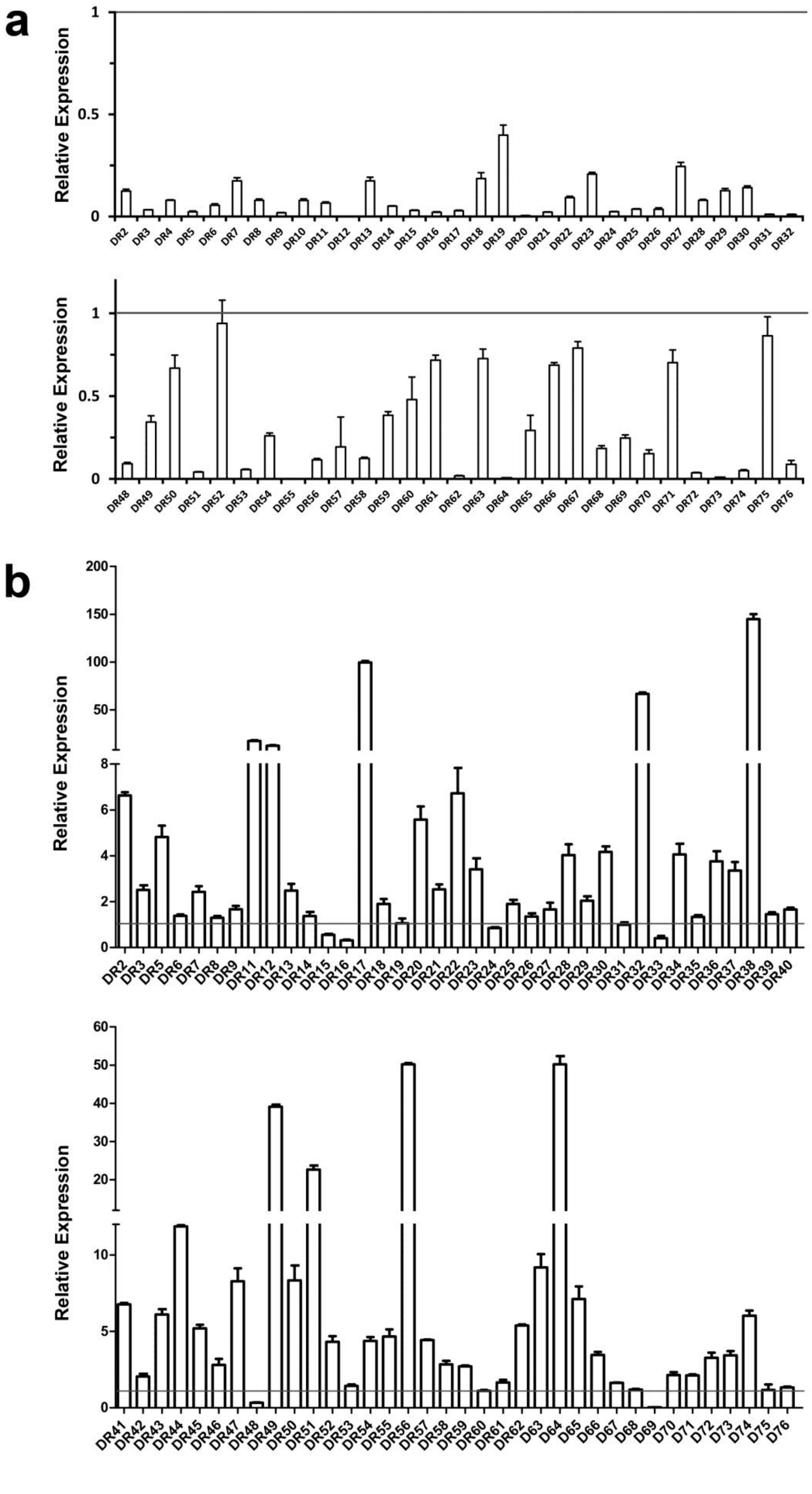
a
Relative Expression
1
0.5
0
DR2 DR3 DR4 DR5 DR6 DR7 DR8 DR9 DR10 DR11 DR12 DR13 DR14 DR15 DR16 DR17 DR18 DR19 DR20 DR21 DR22 DR23 DR24 DR25 DR26 DR27 DR28 DR29 DR30 DR31 DR32
Relative Expression
1
0.5
0
DR48 DR49 DR50 DR51 DR52 DR53 DR54 DR55 DR56 DR57 DR58 DR59 DR60 DR61 DR62 DR63 DR64 DR65 DR66 DR67 DR68 DR69 DR70 DR71 DR72 DR73 DR74 DR75 DR76
b
Relative Expression
200
150
100
50
8
6
4
2
0
DR2 DR3 DR5 DR6 DR7 DR8 DR9 DR11 DR12 DR13 DR14 DR15 DR16 DR17 DR18 DR19 DR20 DR21 DR22 DR23 DR24 DR25 DR26 DR27 DR28 DR29 DR30 DR31 DR32 DR33 DR34 DR35 DR36 DR37 DR38 DR39 DR40
Relative Expression
60
50
40
30
20
10
5
0
DR41 DR42 DR43 DR44 DR45 DR46 DR47 DR48 DR49 DR50 DR51 DR52 DR53 DR54 DR55 DR56 DR57 DR58 DR59 DR60 DR61 DR62 D63 D64 D65 D66 D67 D68 D69 D70 D71 D72 D73 D74 D75 D76

Supplementary Figure 9 Identification of differentially expressed genes in *GhHOX3* co-suppression (line 5-8) and wild-type plants by qRT-PCR. Cotton fibers collected at 6 DPA were used for gene expression analysis.

Genes down-regulated in co-suppression line are shown in **(a)**, and up-regulated in over-expression line in **(b)**. The horizontal line indicates the expression level (normalized to 1) of each gene in wild-type *G. hirsutum* cv. R15. For gene identity, see **Supplementary Table 5**. Data are shown as mean ± s.e.m. (n=3). For expression of *GhRDL1* (DR77, GenBank: AY072821) and *GhEXPA1* (DR78, GenBank: AF512539), see **Fig. 2a-d**.

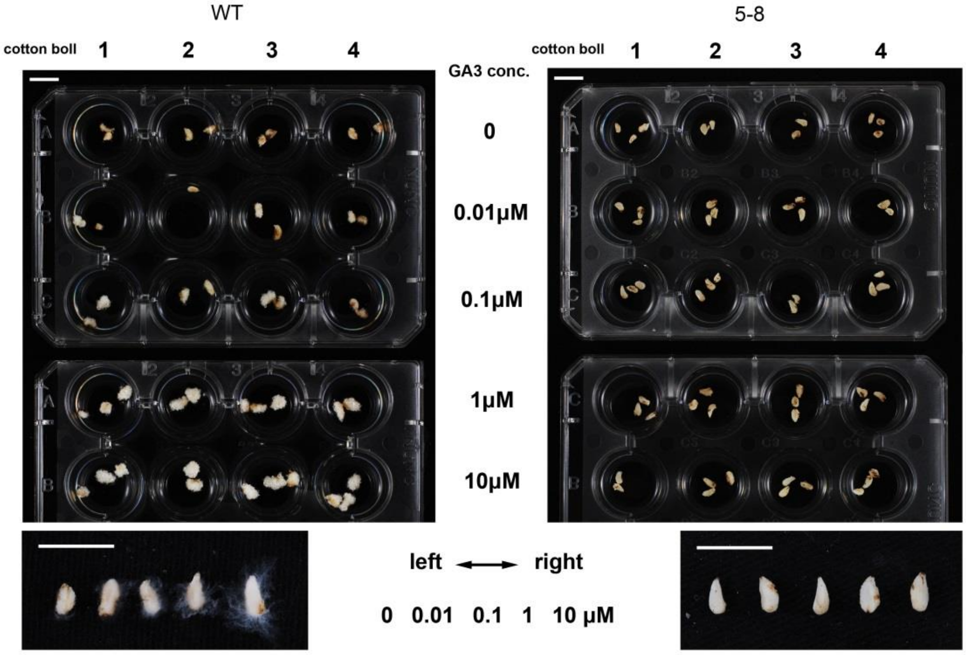

Supplementary Figure 10 Effects of gibberellin (GA) on cotton fiber elongation.

Cotton ovules of wild-type (WT) and *GhHOX3* co-suppression line 5-8 were cultured *in vitro* under gradient concentrations (0~10 μM) of gibberellic acid (GA_3) and a constant concentration (1 μM) of auxin, indole-3-acetic acid (IAA). The ovules harvested at 2 DPA were used a 6-day culture. Numbers on the top indicate ovules from the same cotton boll. Magnified view in bottom panel shows fibers elongated in ovules of the wild-type (left), but not the co-suppression line 5-8 (right). Scale bars, 1cm.

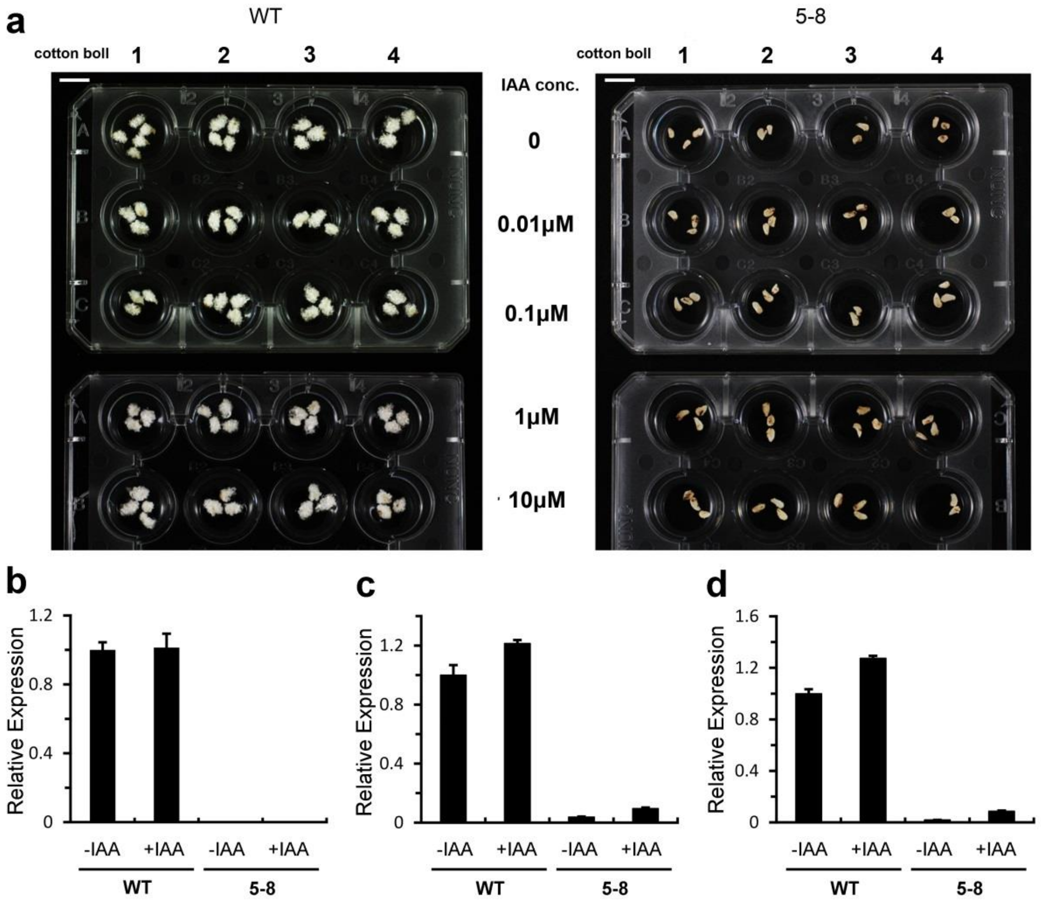

Supplementary Figure 11 Effects of auxin on cotton fiber elongation.

(a) Cotton ovules of wild-type (WT) and *GhHOX3* co-suppression line 5-8 were cultured *in vitro* under gradient concentrations (0~10 μM) of auxin and a constant concentration (1 μM) of GA. The ovules harvested at 2 DPA were used for a 6-day culture. Numbers on the top indicate ovules from the same cotton boll. Scale bars, 1cm. **(b-d)** Expression level of *GhHOX3* **(b)** and its down-stream target genes *GhRDL1* **(c)** and *GhEXPA1* **(d)**. Data are shown as mean ± s.e.m. (n=3).

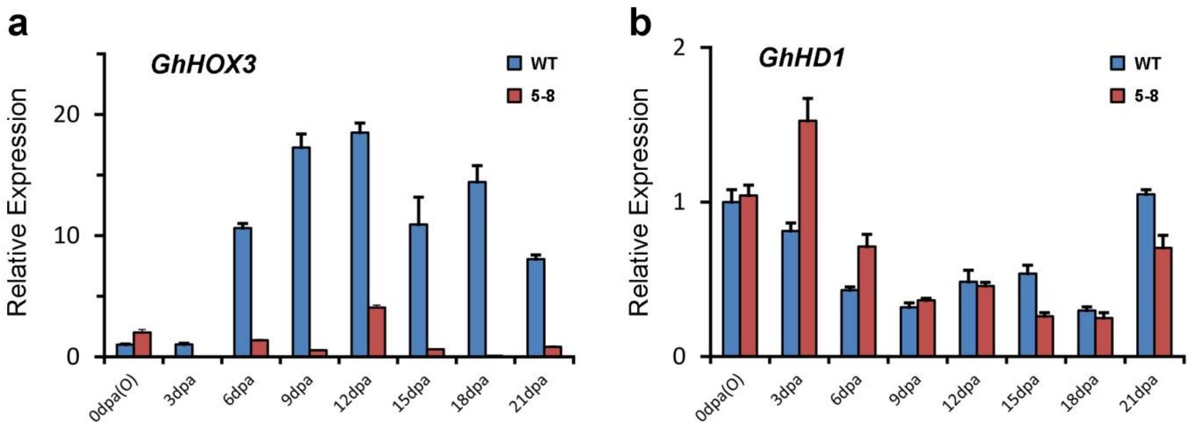

Supplementary Figure 12 Expression of *GhHD1* (GenBank: DQ006269) in co-suppression line 5-8.

(a,b) Transcript levels of *GhHOX3* **(a)** and *GhHD1* **(b)** in line 5-8 and wild-type (WT) were detected by qRT-PCR. Cotton fiber or ovule (O) were harvested at different DPA as indicated. Data are shown as mean ± s.e.m. (n=3).

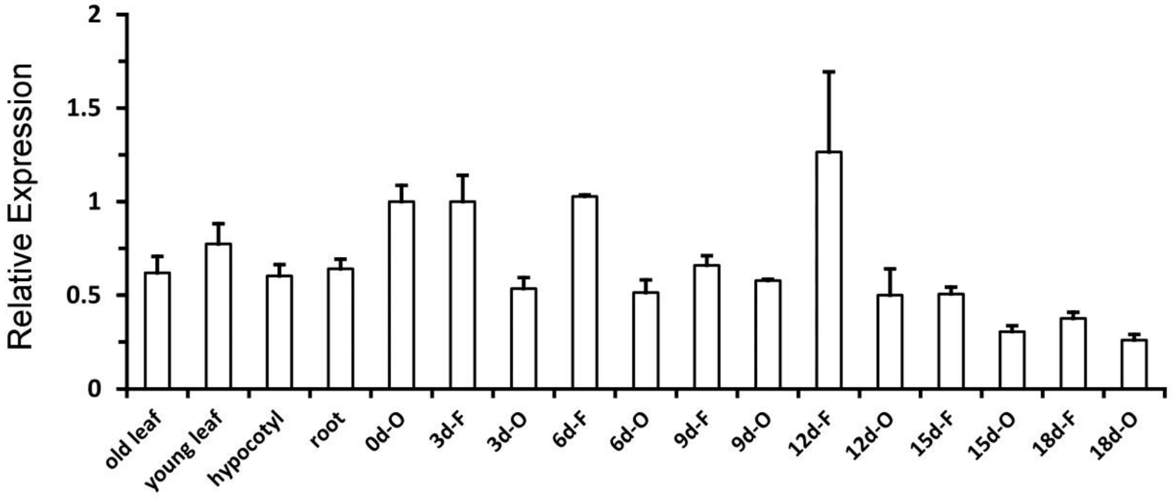

Supplementary Figure 13 Expression pattern of *GhSLR1* in cotton (*G. hirsutum*).

Analysis of *GhSLR1* (GenBank: DQ006269) transcript levels in various organs of cotton by qRT-PCR. Old leaf, fully expended leaf from 2-month old plant. Young leaf, not fully expended true leaf from 2-week old plant. Cotton fiber (F) or ovule (O) were harvested at different DPA as indicated. Data are shown as mean ± s.e.m. (n=3).

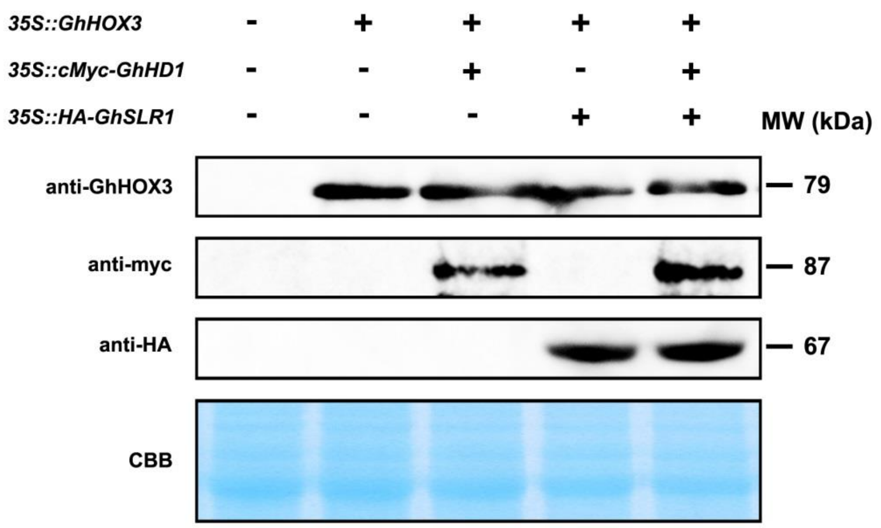

Supplementary Figure 14 Western blot detection of protein amount in co-filtrated tobacco leaves.

GhHOX3, myc, and HA antibody were respectively used for detecting corresponding protein contents, as indicated. CBB: Coomassie Brilliant Blue staining of total protein extracted, as loading control.

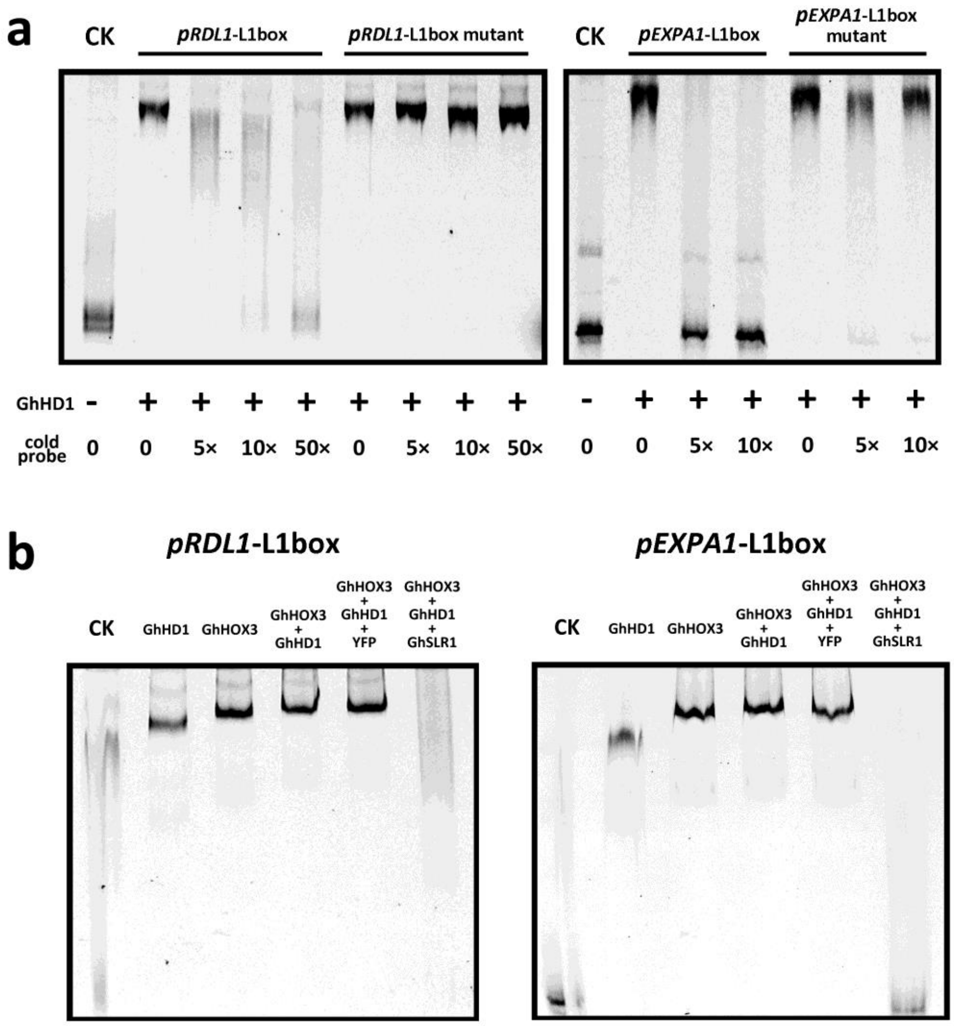

Supplementary Figure 15 EMSA assay of binding of GhHD1 and GhHOX3 to L1-box cis-elements from the *GhRDL1* and *GhEXPA1* promoters.

(a) The 6 x fragments of *GhRDL1* and *GhEXPA1* promoters containing the intact or the mutated L1-box (see Fig. 2e) were used for the assay. Labeled *GhRDL1* and *GhEXPA1* fragments were incubated with 1 μg of MBP-GhHD1 to compete with different concentrations (5×, 10× and 50×) of cold probes of either intact or mutated L1-box. **(b)** Fragments of *GhRDL1* and *GhEXPA1* promoters containing the intact or the mutated L1-box were incubated with the combination of the following proteins: 0.5 μg MBP-GhHD1, 1 μg MBP-GhHOX3, 1 μg GST-YFP (as control) and 1 μg GST-GhSLR1, as indicated on the top. Note that GhSLR1 inhibited the binding of GhHOX3/ GhHD1 to L1-box.

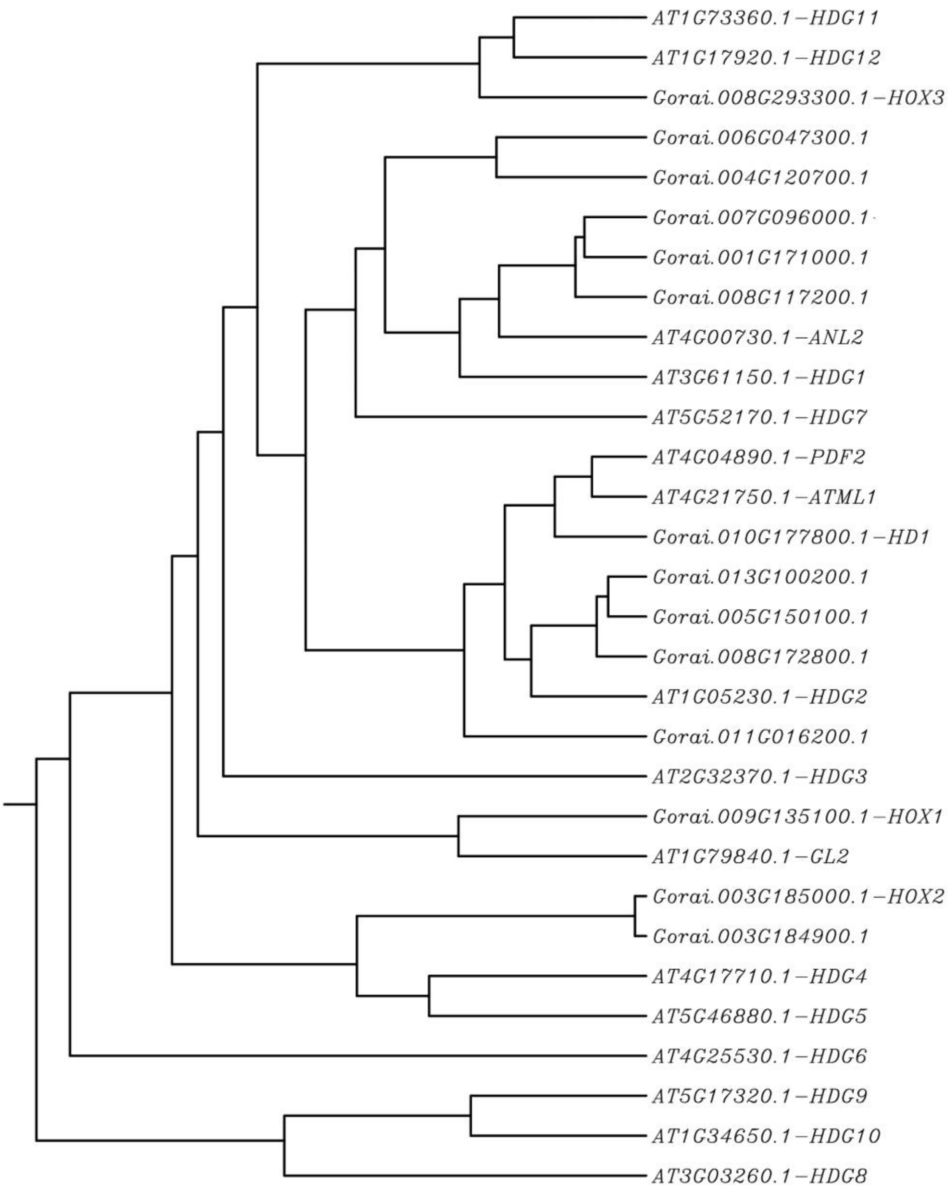

Supplementary Figure 16 Cladogram of HD-ZIP IV subfamily members of *Arabidopsis thaliana* and the sequenced diploid cotton *Gossypium raimondii*. Amino acid sequences of HD-ZIP IV proteins were aligned by the CLUSTAL W (v1.81). Cotton HOX1, HOX2, HOX3 and HD1 are indicated.

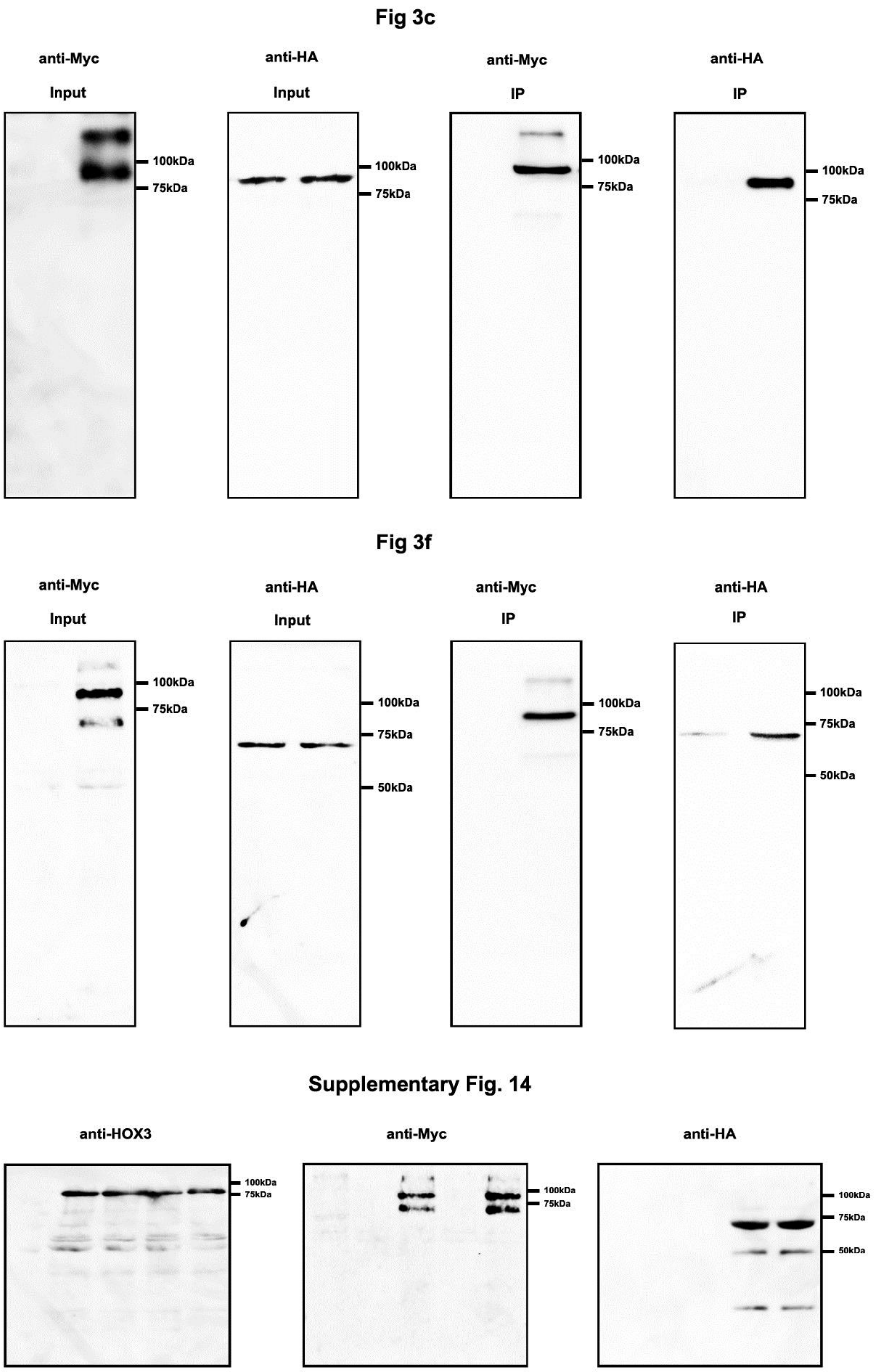

Supplementary Figure 17 The whole view of immunoblot blots partially shown in Fig 3c, 3f and Supplementary Fig 14.

Supplementary Table 1 Integration *GhHOX3* with previously reported QTLs.

Gene	Subgenome	Chromosomal location	Previously reported QTL associated with specific chromosome[a]
GhHOX3	A	A12	FL[1,2,3]; FU[1]; FF[4]
	D	D12	FL[5]; FS[2];

FE, Elongation; FF, fineness; FL, length; FS, strength; FU, uniformity

Supplementary Table 2 Association analysis of fiber qualities and SNPs in *GhHOX3-A* (located in chromosome A12, Chr.12).

**Fiber quality**	**C(198)**	**T(83)**	**Difference**	**P value**	**G(100)**	**A(81)**	**Difference**	**P value**
Strength (cN/tex)	28.93	28.13	0.80**	2.329E-36	28.92	28.16	0.76**	1.205E-32
Length (mm)	29.08	28.74	0.34**	1.682E-13	29.10	28.69	0.41**	1.885E-18
Micronaire	4.48	4.46	0.02	0.1363	4.48	4.48	0.00	0.8257
Uniformity Ratio %	84.09	83.76	0.33**	8.965E-19	84.08	83.78	0.30**	3.008E-16

Data of fiber qualities of 281 cultivars or lines of Upland cotton (*G. hirsutum*). The number of cultivars/lines analyzed are indicated in bracket. A, adenine; C, cytosine; G, guanine; T, guanine. Data are shown in student's *t*-test (* P <0.05; ** P<0.01).

Supplementary Table 3 Fiber length and 100-seed-weight of T_0 plants of *35S::GhHOX3* transgenic lines.

**Number of T_0 transgenic lines (R15 as the WT control)**	**Fiber length (mm)**	**Weight of 100 seeds (g)**
#1	26.03±1.21	10.10
#2	9.67±1.48**	10.23
#3	10.04±1.42**	10.76
#4	14.55±1.98**	9.04
#5	10.34±1.25**	8.92
#6	10.82±1.09**	12.25
WT	25.26±1.00	10.36

Data are given as mean ± s.e.m., and analyzed in a student's *t*-test (*P < 0.05; **P < 0.01) compared to wild-type *G. hirsutum* cv. R15 (WT).

Supplementary Table 4 Deduced GhHOX3 target genes containing L1-box in promoter.

Custom ID	Gene Annotation (BLAST nr)	L1-box location
DR2	gi|224064362|ref|XP_002301438.1|/7.03976e-13/predicted protein [*Populus trichocarpa*]	-1751
DR11	gi|224085499|ref|XP_002307596.1|/1.34009e-34/predicted protein [*Populus trichocarpa*]	-1413
DR17	gi|359478710|ref|XP_002282495.2|/4.5478e-63/PREDICTED: bifunctional monodehydroascorbate reductase and carbonic anhydrase nectarin-3-like [*Vitis vinifera*]	-817
DR20	-	-1533
DR30	gi|147785317|emb|CAN72854.1|/8.06842e-17/hypothetical protein VITISV_043216 [*Vitis vinifera*]	-487
DR45	gi|30841320|gb|AAO92740.1|/8.76353e-102/auxin binding protein [*Gossypium hirsutum*]	-330
DR46	gi|188509926|gb|ACD56615.1|/3.50549e-51/predicted protein [*Gossypioides kirkii*]	-253
DR47	-	-1667
DR51	gi|224085499|ref|XP_002307596.1|/6.80547e-34/predicted protein [*Populus trichocarpa*]	-1413
DR54	gi|225432018|ref|XP_002273484.1|/4.4153e-36/PREDICTED: putative invertase inhibitor [*Vitis vinifera*]	-1574
DR55	gi|224142001|ref|XP_002324349.1|/1.50715e-141/predicted protein [*Populus trichocarpa*]	-781
DR56	gi|255572787|ref|XP_002527326.1|/2.56946e-16/Vegetative cell wall protein gp1 precursor, putative [*Ricinus communis*]	-396, -568, -843
DR59	gi|255539022|ref|XP_002510576.1|/3.79077e-50/skp1, putative [Ricinus communis]	-378,-1178
DR70	gi|229893912|gb|ACQ90301.1|/1.53406e-43/squalene epoxidase [*Gynostemma pentaphyllum*]	-172
DR72	gi|133925931|gb|ABO43717.1|/4.94432e-75/profilin [*Gossypium hirsutum*]	-928
DR77	gi|18447761|gb|AAL67991.1|/1.46736e-149/dehydration-induced protein RD22-like protein [*Gossypium hirsutum*]	-221
DR78	gi|324984057|gb|ADY68811.1|/1.14956e-124/alpha-expansin 1 [*Gossypium raimondii*]	-787

L1-box location: the distance (nt) of the L1-box *cis*-element to the coding region.

Supplementary Table 5 Proteins interacting with GhHOX3 identified by yeast-two hybrid screen.

**Putative protein type**	**Average frequency**
Auxin response factor (ARF), auxin signaling regulator	9
TCP family transcription factor	8
CCR4-NOT complex subunit	5
WRKY family transcription factor	3
HD-ZIP IV family transcription factor	3
C3H4-RING family protein	3
Serine acetyltransferase	3
JAZ protein, JA signaling repressor	2
DELLA protein, GA signaling repressor	2
ARR protein, cytokinin signaling regulator	2

Yeast-two hybrid assay was repeated using GhHOX3-A and GhHOX3-D, respectively. Average frequency indicates the average numbers of clones obtained for each protein family.

Supplementary Table 6 Oligonucleotide primers used in this investigation.

Primer	Sequence(5'-3')	Purpose
SNP2560-F	CACCAGTACAACCTGTTCATATCTCTTCATTAGACT	EcoTILLING assay
SNP2560-R1	GATCTGAATTTGGCAACTATCCCCACTTG	EcoTILLING assay
SNP2560-R2	CAACCAGAAGGGAGTCTATGAGATCGACATT	EcoTILLING assay
SNP2761-F	AAATTATTTAGGCCCCAATGTGTGAATAGGTGT	EcoTILLING assay
SNP2761-R1	ATCTCCACATGTTCTAACCAAGTAACCTTGGAGT	EcoTILLING assay
SNP2761-R2	CAACCAGAAGGGAGTCTATGAGATCGACATT	EcoTILLING assay
HOX3-ds1-SacI-F	CGAGCTC GGATGTGCGAATGGTTTGCTTG	dsRNA
HOX3-ds1-NotI-R	ATTTGCGGCC GCGGACTGCGTTGCCGTTGGATAG	dsRNA
HOX3-ds1-SmaI-F	TCCCCCGGG GGATGTGCGAATGGTTTGCTTG	dsRNA
HOX3-ds1-XbaI-R	TGCTCTAGA GGACTGCGTTGCCGTTGGATAG	dsRNA
Kan-F	GGCGATACCGTAAAGCACGAGGAA	Transgene identification
Kan-R	GCTATGACTGGGCACAACAGACAAT	Transgene identification
pGhRDL1-F	CTAGAGTTGCATCATGTTTTAT	Gene cloning
pGhRDL1-R	CTAGAACAGGAGTGACTAATTCT	Gene cloning
pGhEXPA1-F	ATCCCATCAAACCAATATCACC	Gene cloning
pGhEXPA1-R	TTGAGTAAGAGCTAGCTAGCTCA	Gene cloning
pGhHOX3-F	AGGCATATTCATCATTCCCCAC	Gene cloning
pGhHOX3-R	AACGTCTCTCCTTTACGAAACACC	Gene cloning
GhHOX3-cds-F	ATGGATTGCGGAAGCGGCGGC	Gene cloning
GhHOX3-cds-R	AGAACTAGGACAATTCAAAGC	Gene cloning
GhHOX4-cds-F	ATGGGCGTTGACAATAGAACAGCC	Gene cloning
GhHOX4-cds-R	TTACTCAACAGATCCCAGCTGTGTTAC	Gene cloning
GhHOX5-cds-F	ATGAGTTTTGGGGGATTTTTGG	Gene cloning
GhHOX5-cds-R	TCAGCTTTCACATTGAAGGGCAGC	Gene cloning
GhHD1-cds-F	ATGTTTAGCCCCAACTTATTTGAGAGT	Gene cloning
GhHD1-cds-R	CGGTCAAGCATTATTGCACTTTAC	Gene cloning
GhSLR1-cds-F	ATGAAGAGAGATCATCAAG	Gene cloning
GhSLR1-cds-R	ACTCAGCTCCTGAGTTAACTCACT	Gene cloning
GhHOX3-KpnI-F	GGGGTACC ATGGATTGCGGAAGCGGC	BiFc
GhHOX3-BamHI-R	CGGGATCC AGAACTAGGACAATTCAAAGC	BiFc
GhHD1-KpnI-F	GGGGTACC ATGTTTAGCCCCAACTTATTTGAG	BiFc
GhHD1-SalI-R	GCGTCGAC TCAAGCATTATTGCACTTTACGGC	BiFc
GhSLR1-KpnI-F	GGGGTACC ATGAAGAGAGATCATCAAG	BiFc
GhSLR1-SalI-A	GCGTCGAC ACTCAGCTCCTGAGTTAACTCACT	BiFc
GhHOX3-NdeI-F	CGCCATATG ATGGATTGCGGAAGCGGCGGC	Yeast hybrid
GhHOX3-SmaI-R	TCCCCCGGG AGAACTAGGACAATTCAAAGC	Yeast hybrid
GhHD1-NdeI-S	CCCATATG ATGTTTAGCCCCAACTTATTTGAG	Yeast hybrid
GhHD1-SmaI-A	TCCCCCGGG TCAAGCATTATTGCACTTTACGGC	Yeast hybrid
GhHD1-SmaI-S	TCCCCCGGG G ATGTTTAGCCCCAACTTATTTGAG	Yeast hybrid
GhHD1-SalI-A	GCGTCGAC TCAAGCATTATTGCACTTTACGGC	Yeast hybrid
Histone3-F	GGCATACCTTGTGGGTCTTTTTGA	qRT-PCR

Primer	Sequence(5'-3')	Purpose
Histone3-R	CTACCACTACCATCATGGC	qRT-PCR
GhHOX1-RT-F	ATATTACCTTCAGGCTTCTC	qRT-PCR
GhHOX1-RT-R	TCACCCATCTTCACATTGCA	qRT-PCR
GhHOX2-RT-F	GAAGCAAATGGGCATAATAT	qRT-PCR
GhHOX2-RT-R	CTATTTTTCTTTACCATTGT	qRT-PCR
GhHOX3-RT-F	ATTATGAATGGCTTAGCTTTGG	qRT-PCR
GhHOX3-RT-R	ACTGCGTTGCCGTTGGATAG	qRT-PCR
GhHD1-RT-F	GTTATGACCCGAAAGAGCA	qRT-PCR
GhHD1-RT-R	CATCCCTTAGGAAATCAAATAC	qRT-PCR
GhSLR1-RT-F	TGAACAAGAAGCGAATCACAACGGT	qRT-PCR
GhSLR1-RT-R	TAAGTATAACTCGGACATAGCCAGG	qRT-PCR
GhRDL1-RT-F	ATACCGTTTTCATCTGACAAGTTGC	qRT-PCR
GhRDL1-RT-R	CAGCTGCTATTGTATACTTTTGCA	qRT-PCR
GhEXPA1-RT-F	GCAGGACTATCACAGCCTACAA	qRT-PCR
GhEXPA1-RT-R	ATGGCACTTGCTCGCCTATTT	qRT-PCR
GhEXPA2-RT-F	CAACATGGTGTTGATAACCG	qRT-PCR
GhEXPA2-RT-R	AGTTTGGTTCACTATTAGG	qRT-PCR
DR2-RT-F	GACGACGAAGAGCAGAGAA	qRT-PCR
DR2-RT-R	ACGATCAAATGACCAAGTG	qRT-PCR
DR3-RT-F	TTGGTTCCAATGTGGTGTTT	qRT-PCR
DR3-RT-R	TTTCGTTCCCCATATGTTCT	qRT-PCR
DR5-RT-F	CTGCCTAACTGTTGCCTCAC	qRT-PCR
DR5-RT-R	TATTCACTCCTCATCCTGGA	qRT-PCR
DR6-RT-F	TCTCAAAAGTATTGGTCATAT	qRT-PCR
DR6-RT-R	TTCTCTTCTTCCGTAAAGTGG	qRT-PCR
DR7-RT-F	AGCACAGTCGCTAGAAGCGGT	qRT-PCR
DR7-RT-R	GACACAAGGGAAGAAAAAATC	qRT-PCR
DR8-RT-F	AAGGAGAAAATGAAGAAGATG	qRT-PCR
DR8-RT-R	AAACCCAGGAGAAACAAGAAG	qRT-PCR
DR9-RT-F	CCTTACAACGAACCGTCCCAC	qRT-PCR
DR9-RT-R	CATTCTCCGCTTTCCCACCCC	qRT-PCR
DR10-RT-F	AGACAAACAAAACAGGAATAA	qRT-PCR
DR10-RT-R	ACAGTGAAAGAGGAAAAGAAA	qRT-PCR
DR11-RT-F	ACGGTGGTGCTTCATCACTTG	qRT-PCR
DR11-RT-R	TTGTTGGAACATTACTCTTTG	qRT-PCR
DR12-RT-F	TGTTGGGTTTTTCAAGCATGG	qRT-PCR
DR12-RT-R	AATTGGTAATCTGGGAAGTGG	qRT-PCR
DR13-RT-F	TGCTATCGCCTTTCCATCTCTG	qRT-PCR
DR13-RT-R	GATCAATTTCATCCTGTTCGGG	qRT-PCR
DR15-RT-F	TGAGAATAATGCGACGAAGCCA	qRT-PCR
DR15-RT-R	CTCCATAATCCAAGGGACCAGA	qRT-PCR
DR16-RT-F	GCAGCAAGCCGGCAGCCGGGAC	qRT-PCR
DR16-RT-R	CATCTGTATGATTTAAGAACAG	qRT-PCR

Primer	Sequence(5'-3')	Purpose
DR17-RT-F	CAGCGACGGACGAAAGCAATCT	qRT-PCR
DR17-RT-R	CCCTGACCATACCACCGCAACA	qRT-PCR
DR18-RT-F	AAGATGTGTGGATATGAAGACG	qRT-PCR
DR18-RT-R	AAATGAAGATGATGAGGCAGTA	qRT-PCR
DR19-RT-F	ATAGCCACCGTCACCTTCGCAG	qRT-PCR
DR19-RT-R	TAATAGATTATCCAGAACCCCA	qRT-PCR
DR20-RT-F	GATGGTAGATCTAGGATTGAAG	qRT-PCR
DR20-RT-R	GCCATTTGCATATTTACCGGTA	qRT-PCR
DR21-RT-F	AGATGACAAACGAGACACCACA	qRT-PCR
DR21-RT-R	TCTTCCGCACAAATACAGCCCA	qRT-PCR
DR23-RT-F	GGGAAACAGCAACACCATGATC	qRT-PCR
DR23-RT-R	TAGTGGCCACAGGTGCAGTCTG	qRT-PCR
DR24-RT-F	ACACATCCGCACACAACCCCTG	qRT-PCR
DR24-RT-R	GGAACTGCCATCACCACTTCAC	qRT-PCR
DR25-RT-F	AAAGCCGACAAAAATCAGAGAA	qRT-PCR
DR25-RT-R	CAAACCACAAAGAGAGGGGACA	qRT-PCR
DR26-RT-F	CTTCAACCCTGGCCTACTCTCC	qRT-PCR
DR26-RT-R	CCAAGCATCACTCCGTTTCTTC	qRT-PCR
DR27-RT-F	TGAAGAGTTATAGAGGGGTGAG	qRT-PCR
DR27-RT-R	TTCGATGGGGAAATTGAGAGTA	qRT-PCR
DR28-RT-F	GTCGTCAAAGACTCGGGAATCG	qRT-PCR
DR28-RT-R	ACCCTGGCTATGAAATCATCAG	qRT-PCR
DR29-RT-F	GCCATATCCCCAGTATCAAACA	qRT-PCR
DR29-RT-R	TTCAAAATCAAACCATCCCATT	qRT-PCR
DR30-RT-F	AAAGAAAACAAGATCCCAGCAG	qRT-PCR
DR30-RT-R	TAGAGTGAGAATCATCAAAAGC	qRT-PCR
DR32-RT-F	TGCCTCTCATTTATTACCGACG	qRT-PCR
DR32-RT-R	AGACCCAACCACCATTCCAACC	qRT-PCR
DR33-RT-F	CATACAAGGATGGTGGAGAAAT	qRT-PCR
DR33-RT-R	GCAGCTCAGGATCAGTAAAACT	qRT-PCR
DR34-RT-F	TAAAGACGGGGAATGGGTGGAT	qRT-PCR
DR34-RT-R	TCGCTGCCGGGGTTGTAAAACG	qRT-PCR
DR35-RT-F	AATGCCAGTTATTATCGGCCGA	qRT-PCR
DR35-RT-R	AACAATTGAGAGACGAAGGTTC	qRT-PCR
DR37-RT-F	ATTCTCAGCAAGTGTCCGTCAA	qRT-PCR
DR37-RT-R	ATCCCTAATCCTCAGCATCCCC	qRT-PCR
DR38-RT-F	GTCTCATACCTGGATTATTTGG	qRT-PCR
DR38-RT-R	GAAGCATTCTCTCCCTCGCCTT	qRT-PCR
DR39-RT-F	TTACTCCGGCCTCGCTGCCACA	qRT-PCR
DR39-RT-R	TTGAATAACAACAAGGACTTCA	qRT-PCR
DR40-RT-F	CAAGGACAAGGCAGTCACAGGA	qRT-PCR
DR40-RT-R	GGGTCGAAGTTGGACACACCAA	qRT-PCR
DR41-RT-F	AGAAATACAAACACTTCGTCAA	qRT-PCR

Primer	Sequence(5'-3')	Purpose
DR41-RT-R	TAGTTCCTCAGCCTATGCTCAC	qRT-PCR
DR42-RT-F	ATTGCACCGACTTGATCTTAGC	qRT-PCR
DR42-RT-R	TATTTTGTTCCATTATTTTCCT	qRT-PCR
DR44-RT-F	TTGTCGCTTTGTTCTTCCATCC	qRT-PCR
DR44-RT-R	TGCCTTCCATTATTAGCCTTCG	qRT-PCR
DR45-RT-F	CAGCCGACTTCTGTGTTGGGGA	qRT-PCR
DR45-RT-R	CTGGTGTTATTGCAGCTTTTAC	qRT-PCR
DR46-RT-F	TATTGGGGTTTGCCCTTTGTGC	qRT-PCR
DR46-RT-R	GGGAGCTTGTTGATGGGTGGTT	qRT-PCR
DR47-RT-F	AGGGGTCAAGTTAAGATGAGAA	qRT-PCR
DR47-RT-R	CAGAAAGGTAGCCCGTTGGGGT	qRT-PCR
DR48-RT-F	CAAAACTACAAACCATCATTCG	qRT-PCR
DR48-RT-R	AGCAACATCATCCATAAACCTA	qRT-PCR
DR49-RT-F	AGTTCGACACGTCTTTCTTCAC	qRT-PCR
DR49-RT-R	TACCCGTCTTCACACCTATATT	qRT-PCR
DR50-RT-F	GTTGATATCCCCAACACCAACA	qRT-PCR
DR50-RT-R	TCACAAACACCAGCAGTGCCTC	qRT-PCR
DR52-RT-F	GAAGAAGATGATGCTGGTTATG	qRT-PCR
DR52-RT-R	CTCGTTGTAGGGGGAGGAGGTGG	qRT-PCR
DR53-RT-F	CTTTCGCCTCTCTTCTTTACCA	qRT-PCR
DR53-RT-R	AAACCCATCACTTTCATCCTCA	qRT-PCR
DR55-RT-F	GTCGCCTCTCTTTTACCCTCTT	qRT-PCR
DR55-RT-R	ATTGACTTCACCCTTACTTCGT	qRT-PCR
DR57-RT-F	ATGCCTTCCCTCCTTGCCAAAC	qRT-PCR
DR57-RT-R	ACTCATTCTTCCAATAATACCC	qRT-PCR
DR58-RT-F	GAGTTGAACATGGGGTTAAAAG	qRT-PCR
DR58-RT-R	CAAGCAGAGAAAGAGTAGAGGG	qRT-PCR
DR60-RT-F	GCTTCAATCGATAATACTACCG	qRT-PCR
DR60-RT-R	TGTTTCAGCCTCATTTCCACCA	qRT-PCR
DR61-RT-F	GCTCCTGTTGCTGACCCAAACC	qRT-PCR
DR61-RT-R	CTGATCCATTATCGACAATCTC	qRT-PCR
DR62-RT-F	CTAACTCCACCATCGTCCACAG	qRT-PCR
DR62-RT-R	ACTTGAAAATCACAAATCCCAA	qRT-PCR
DR63-RT-F	ATGGGAAAGGAGATGAGGCATT	qRT-PCR
DR63-RT-R	CCAACACTGGAGAATTAGATGT	qRT-PCR
DR64-RT-F	GCCTCCCACGCTGCCTTCCTTT	qRT-PCR
DR64-RT-R	ATGTATCACCACAGAGCGGTTC	qRT-PCR
DR65-RT-F	TGTTCTTTACATCTGCTTTGGA	qRT-PCR
DR65-RT-R	AACCTTGCCCGTTTTTGTCCTC	qRT-PCR
DR66-RT-F	TACTCTTTAAAATCTCAGTAGC	qRT-PCR
DR66-RT-R	TGTAGAAGTTGAAGGAGGTCCG	qRT-PCR
DR68-RT-F	GTCGGATTGGGGTCCCGTTTTC	qRT-PCR
DR68-RT-R	CAAATGAACCTTAACCGCCAGC	qRT-PCR

Primer	Sequence(5'-3')	Purpose
DR69-RT-F	ATGGTCGATAGTGGATCCGAGC	qRT-PCR
DR69-RT-R	ACACAAAAGGCAAAAAGTGAGG	qRT-PCR
DR70-RT-F	CAAAGACTACGAAAGAAAGCCG	qRT-PCR
DR70-RT-R	ATCACAGACGATGGTGAGAGGA	qRT-PCR
DR71-RT-F	GCTTTGCTCCCTTGTTCTTATT	qRT-PCR
DR71-RT-R	ACACCTGTTGCCCAGGTTGTCT	qRT-PCR
DR72-RT-F	TGGGCTCAGAGCTCTAACTTCC	qRT-PCR
DR72-RT-R	CACCCCTCCAGATCCCTTTTTT	qRT-PCR
DR73-RT-F	GAAGATAAAATACCCAAACCCT	qRT-PCR
DR73-RT-R	TCGAATCGACCGAAACCAAGAT	qRT-PCR
DR74-RT-F	TTACATCCCTCGATCACCGCCT	qRT-PCR
DR74-RT-R	TCCACTAACAATCACTGCCACA	qRT-PCR
DR75-RT-F	ACACATCACATTACGACTCTGC	qRT-PCR
DR75-RT-R	TGTTTCTGTACCTTATTGCCCC	qRT-PCR
DR76-RT-F	ATCATGCGGTGTATCGATGTCC	qRT-PCR
DR76-RT-R	CAGCTTCTCAATCTCCTTGGCG	qRT-PCR

Supplementary References

1 Luan, M., Guo, X., Zhang, Y., Yao, J. & Chen, W. QTL mapping for agronomic and fibre traits using two interspecific chromosome substitution lines of Upland cotton. *Plant breeding* **128**, 671-679 (2009).

2 Lin, Z.-X. *et al.* Linkage map construction and mapping QTL for cotton fibre quality using SRAP, SSR and RAPD. *Plant breeding* **124**, 180-187 (2005).

3 Qin, H., Guo, W., Zhang, Y.-M. & Zhang, T. QTL mapping of yield and fiber traits based on a four-way cross population in *Gossypium hirsutum* L. *Theoretical and Applied Genetics* **117**, 883-894 (2008).

4 Shen, X. *et al.* Molecular mapping of QTLs for fiber qualities in three diverse lines in Upland cotton using SSR markers. *Molecular breeding* **15**, 169-181 (2005).

5 He, D.-H. *et al.* QTL mapping for economic traits based on a dense genetic map of cotton with PCR-based markers using the interspecific cross of *Gossypium hirsutum* × *Gossypium barbadense*. *Euphytica* **153**, 181-197 (2007).
